# A Computational Assay of Estrogen Receptor α Antagonists Reveals the Key Common Structural Traits of Drugs Effectively Fighting Refractory Breast Cancers

**DOI:** 10.1038/s41598-017-17364-4

**Published:** 2018-01-12

**Authors:** Matic Pavlin, Angelo Spinello, Marzia Pennati, Nadia Zaffaroni, Silvia Gobbi, Alessandra Bisi, Giorgio Colombo, Alessandra Magistrato

**Affiliations:** 1 CNR-IOM-Democritos c/o International School for Advanced Studies (SISSA), via Bonomea 265, 34136 Trieste, Italy; 2 Fondazione IRCSS Istituto Nazionale dei Tumori, via Amadeo 42, 20113 Milano, Italy; 3 Department of Pharmacy and Biotechnology, Alma Mater Studiorum-University of Bologna, via Belmeloro 6, 40126 Bologna, Italy; 4 CNR-ICRM, via Mario Bianco 9, 20131 Milano, Italy

## Abstract

Somatic mutations of the Estrogen Receptor α (ERα) occur with an up to 40% incidence in ER sensitive breast cancer (BC) patients undergoing prolonged endocrine treatments. These polymorphisms are implicated in acquired resistance, disease relapse, and increased mortality rates, hence representing a current major clinical challenge. Here, multi-microseconds (12.5 µs) molecular dynamics simulations revealed that recurrent ERα polymorphisms (*i*. *e*. L536Q, Y537S, Y537N, D538G) (mERα) are constitutively active in their *apo* form and that they prompt the selection of an agonist (active)-like conformation even upon antagonists binding. Interestingly, our simulations rationalize, for the *first time*, the efficacy profile of (pre)clinically used Selective Estrogen Receptor Modulators/Downregulators (SERMs/SERDs) against these variants, enlightening, at atomistic level of detail, the key common structural traits needed by drugs able to effectively fight refractory BC types. This knowledge represents a key advancement for mechanism-based therapeutics targeting resistant ERα isoforms, potentially allowing the community to move a step closer to ‘precision medicine’ calibrated on patients’ genetic profiles and disease progression.

## Introduction

Breast Cancer (BC) is the most frequent cancer type as well as the second leading cause of death in women. Approximately 70% of BC cases, detected after menopause, express Estrogen Receptor (ER) and are classified as ER sensitive (ER+)^1^. After the menopause, estrogens (17-β-estradiol or estrone) are primarily produced by human aromatase (HA) enzyme, whose deregulated activity leads to increased estrogens levels in malignant tissues^1–5^. These hormones bind as agonist to ERα, which, then, exerts a pro-oncogenic effect by either decreasing apoptosis or promoting cell proliferation^1^. So far, endocrine treatments of ER + BC rely on estrogens deprivation, by inhibiting HA, or on small molecule regulators of ERα, such as selective ERα modulators (SERMs) or downregulators (SERDs). The latter, besides being ERα antagonists, occupies the substrate binding site and induces a conformational change in the receptor, as SERMs do, also prompt ERα ubiquitination and degradation^6^.

**Figure 1:**
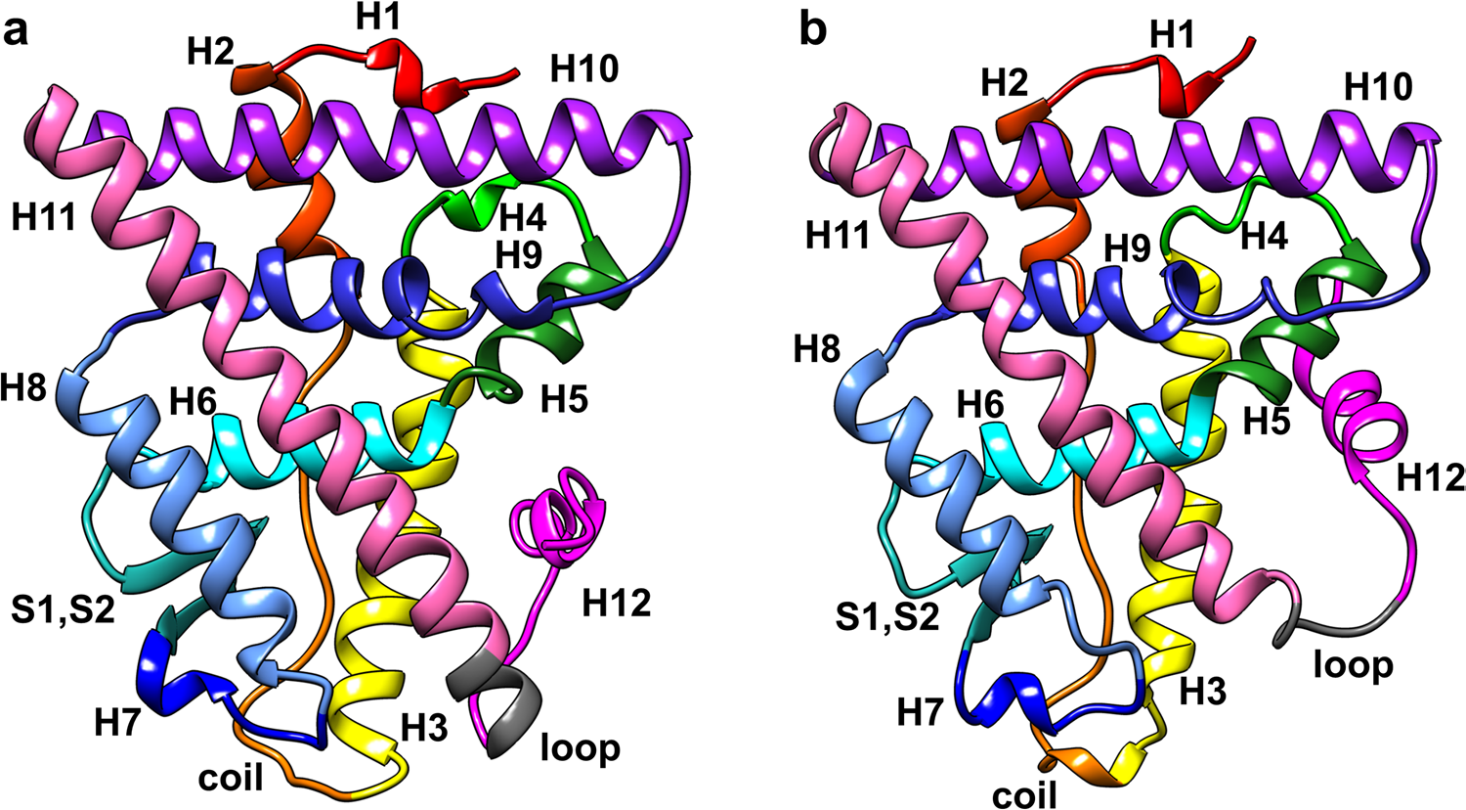
ERα LBD monomer in agonist (**a**) and antagonist conformation (**b**), with secondary structure elements coloured in distinct colours. The helices label comprises both residues forming helices and those located on the loops connecting them. Namely, H1: residues 303–311 (red); H2: residues 312–322 (dark orange); coil2–3 (C2–3): residues 323–338 (orange); H3: residues 339–363 (yellow); H4: residues 364–371 (light green); H5: residues 372–382 (dark green); H6: residues 383–396 (turquoise); S1, S2: residues 397–411 (dark turquoise); H7: residues 412–419 (dark blue); H8: residues 420–439 (light blue); H9: residues 440–463 (blue); H10: residues 464–494 (violet); H11: residues 495–531 (pink and grey; coloured in grey are residues 527–531); H12: residues 532–552 (magenta).

ERα is a nuclear hormone receptor and a ligand-regulated transcription factor, mediating the activity of estrogens in many important physiological processes (*i*. *e*. reproduction, cardiovascular maintenance, bone density/remodelling)^7,8^. ERα is composed by five distinct functional domains (Figure (Fig.) S1 of the Supplementary Information (SI)), among which only the structures of the DNA-binding domain and the ligand-binding domain (LBD) have been determined crystallographically. At physiological conditions ERα exists as a dimer, stabilized by the binding of either agonists or antagonists. The crystal structures have revealed that the most important structural element of each LBD monomer (Fig. 1) is helix 12 (H12), which acts as a molecular switch between the active and inactive conformation of the receptor^9^. Indeed, upon estrogen binding, H12 occludes the ligand-binding site, packing against helixes H3, H5/6, and H11 (Fig. 1a)^10^. This corresponds to the agonist (active) conformation of ERα. In contrast, when an antagonist binds, it prevents H12 from assuming the agonist conformation, and H12 moves towards a groove formed by H3 and H5^10^. This corresponds to the antagonist (inactive) conformation of ERα (Fig. 1b)^11^.

**Figure 2:**
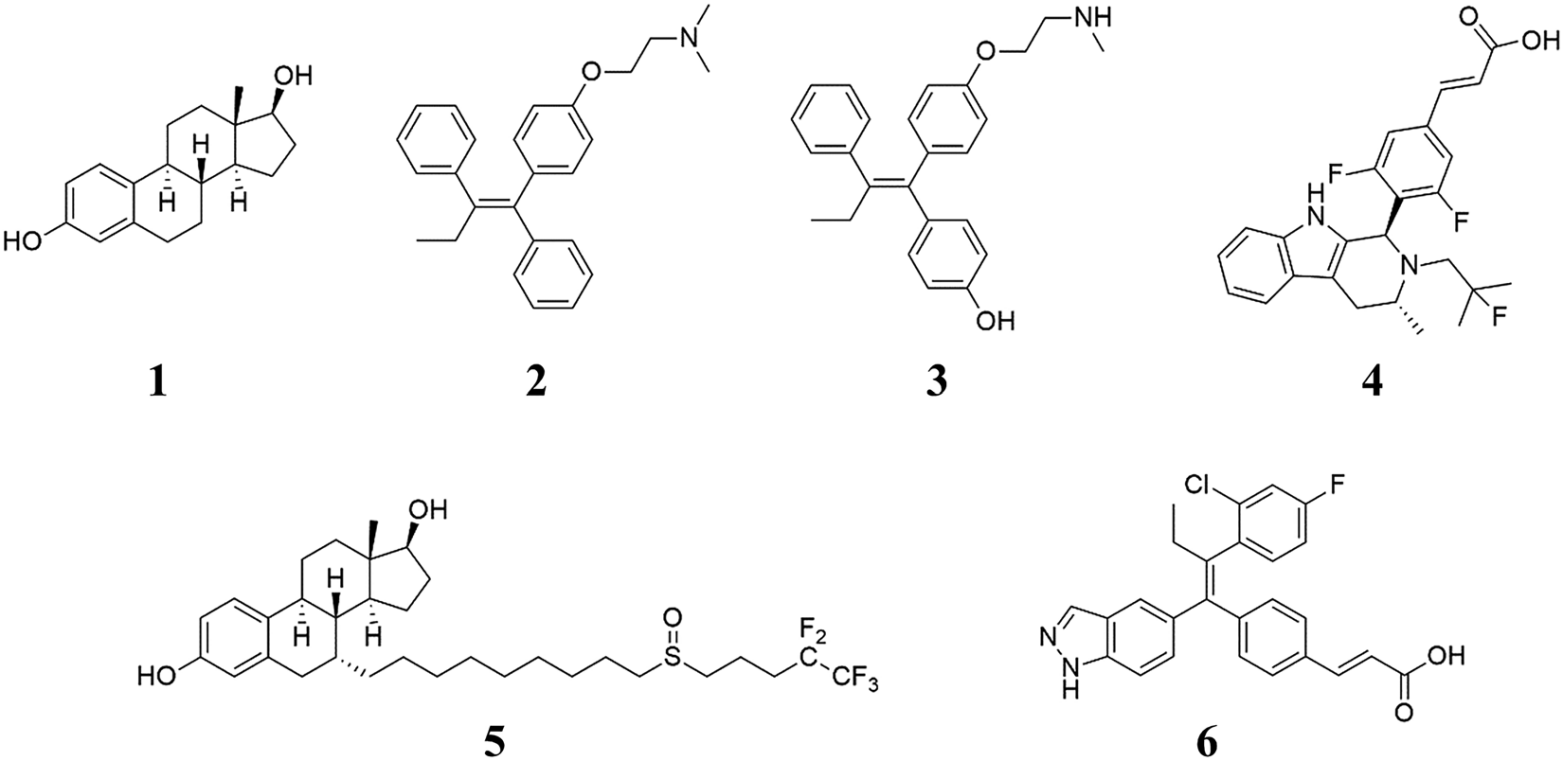
Chemical structures of 17-β-estradiol (**1**), tamoxifen (**2**), endoxifen (**3**), AZD-9496 (**4**), fulvestrant (**5**), and GDC-0810 (**6**).

The most effective ERα antagonists in current clinical use are (Fig. 2): (i) tamoxifen, a SERM, which is active through its metabolites, but it is endowed with agonist effects in peripheral tissues; and (ii) fulvestrant, a SERD downregulating ERα, with no agonist effects^8,12^, which suffers, however, from poor pharmacokinetic properties (*e*. *g*. low solubility in water).

The clinical use of tamoxifen has been extremely beneficial for BC patients in the last decades, reducing BC mortality rates by 25–30%. However, up to 40% of all ER+ BC patients, initially responding to the endocrine treatments, acquire resistance during disease progress and frequently relapse after prolonged therapies. Recent studies by Robinson *et al*.^13^, Toy *et al*.^14^, Merenbakh-Lamin *et al*.^15^, and Jeselsohn *et al*.^16^ pinpointed several ERα polymorphisms (mERαs) located in the LBD, either in the vicinity of the estrogens binding site (*i*. *e*. E380Q), between H9 and H10 (S463P) or, more frequently, in the loop connecting H11 and H12 (*i*. *e*. L536Q, L536R, Y537C, Y537N, Y537S, and D538G). These occur at high rate in relapsed metastatic patients, while being rare or absent in primary tumours of untreated patients^13,14,16^. Independent studies unambiguously demonstrated that these mERαs contributed to the acquired resistance to endocrine treatments^1^, indicating that BC evolves new traits to escape the pressure of currently used therapeutics. The reported frequencies of specific ERα polymorphisms differ according to the clinical study. Namely, the D538G was present in 21–36% of cases^16–18^, Y537N in 5–33%^16,18^, and Y537S in 13–22%^16–18^, while the other polymorphisms are less frequent^14,18^. In few cases a double D538G and Y537S mutant was also observed^14,17^. The abundance of these mutations is not directly correlated to the overall survival time of patients (D538G 26 months, Y537S 20 months, and 15 months for double mutant Y537S and D538G)^17^, with Y537S being the most aggressive isoform.

Since these polymorphisms are highly recurrent, endocrine resistance in BC is becoming a major clinical problem. Cell lines expressing ERα mutations are insensitive to HA inhibitors, but remain partially sensitive to high doses of current anti-estrogen therapies (*i*. *e*. endoxifen/tamoxifen, fulvestrant), enlightening that ERα-mediated signalling remains important even in advanced metastatic BCs refractory to therapies. Experimental studies, aimed at developing novel SERDs, led to the discovery of AZD-9496^19^ and GDC-0810^7^, currently in phase I and II of clinical trials, respectively, for the treatment of advanced ER+ BC (Fig. 2). Importantly, these drugs partially retain their efficacy even in the resistant cell lines.

Despite the clinical incidence of the problem, at this point, no clear molecular picture for the structural mechanisms underlying the emergence of resistance in ER exists, nor for the improved efficacy profile of the novel generation of SERDs against these aggressive variants.

Even though the multifactorial nature of cancer as a disease, in which many different players concur to determine the positive vs. negative response to drugs, our aim here is to address a specific set of molecular properties linked to resistance BC manifestations and rationalize how to overcome this. Atomistic simulations have the potential to clarify such mechanisms and rationalize the efficacy profile of current drugs in specific polymorphisms at the microscopic level, offering a spatial (atomistic) and temporal (ns) resolution inaccessible, yet complementary, to experimental methods^2,20^. Computational studies aimed at clarifying the mechanism of mERαs have so far been limited to the most frequent Y537S and D538G isoforms^11,14,18,21–23^. They enlightened that these mutants are constitutively active, but with a distinct molecular mechanism^15,22,23^. The other mutants, not yet studied, may also have different activation pathway leading to mutant-dependent responses to therapies^23^.

Here we performed force field-based molecular dynamics (MD) simulations, for a cumulative time of 12.5 µs, on wild type (WT) ERα and four selected mERα variants (L536Q, Y537S, Y537N, and D538G) aiming at (i) clarifying how the complex conformational landscape of these ERα isomorphisms induces estrogen (EST) (hereafter EST refers to 17-β-estradiol) independent ERα activation; and (ii) elucidating which are the key structural signatures that render new-generation hormonal therapies (fulvestrant and AZD-9496) effective on these polymorphisms. This complete and detailed investigation represents a step forward towards precision medicine against refractory BC types. Indeed, although ESR1 mutations are not yet a validated clinical biomarker, they are being currently evaluated as a potential biomarker to guide therapeutic decisions. Thus, targeted therapy directed to the ESR1 mutants is a subject of intense preclinical and early clinical work^24^.

## Results and Discussion

Since a complete structure of the ERα is not available, in this work, we focused on the LBD dimer, as in other computational studies^14,22,25^. For the agonist conformation, we considered the *apo* and EST-bound form of WT ERα and four recurrent mERαs (L536Q, Y537S, Y537N, D538G), localized on the loop L11–12, connecting H11 and H12, and endoxifen-, AZD-9496-, and fulvestrant-bound WT ERα and the same four mERαs for the antagonist conformation. For all systems, we performed 500 ns of MD simulations, although convergence was reached after 200 ns (Fig.s S2 and S3). A discussion of the per-residue RMSF is detailed in the SI (Paragraph S1, Fig.s S4 and S5, respectively).

To verify our results, we compared structures of the highest populated cluster (see Table 1 of SI (Table S1)) of complexes obtained from our simulations with available crystal structures. For the EST-bound complexes this was possible only for WT^26^ and Y537S^27^ mutant and in both cases comparison revealed no major structural differences. Additionally, clusters’ population analysis showed that both EST-bound and *apo* ERα, in all polymorphisms studied (WT and mERαs), are quite rigid.

Since our simulations refer to two different conformational states of ERα (*i*. *e*. open (antagonist) and closed (agonist)) and our simulations’ time-scale (500 ns per system) does not allow to directly observe their inter-conversion, we will analyse the two states separately. In the following paragraphs we will look for a metric allowing us to classify the preferred conformational state of H12, the key structural element of ERα signal transmission.

### Agonist conformation

To identify signatures and metric of ERα activation we have initially compared the cross-correlation matrices of *apo* and EST-bound WT (WT/EST) agonist conformations (Fig.s 3 and S6). These reveal a correlation contact between H12 and H3 in the EST/WT adduct (the active form of the receptor), which is not present in *apo* form (the inactive state). Thus, we consider this correlation contact as the signature of ERα activation. Remarkably, the same correlation contact is also present in all mutant *apo* ERα isoforms, highlighting their constitutively active character. This is in line with experimental findings suggesting that ERα polymorphisms are intrinsically active even in the absence of EST and explains why metastatic BC types developing these ERα isoforms are insensitive to HA inhibitors.

**Figure 3:**
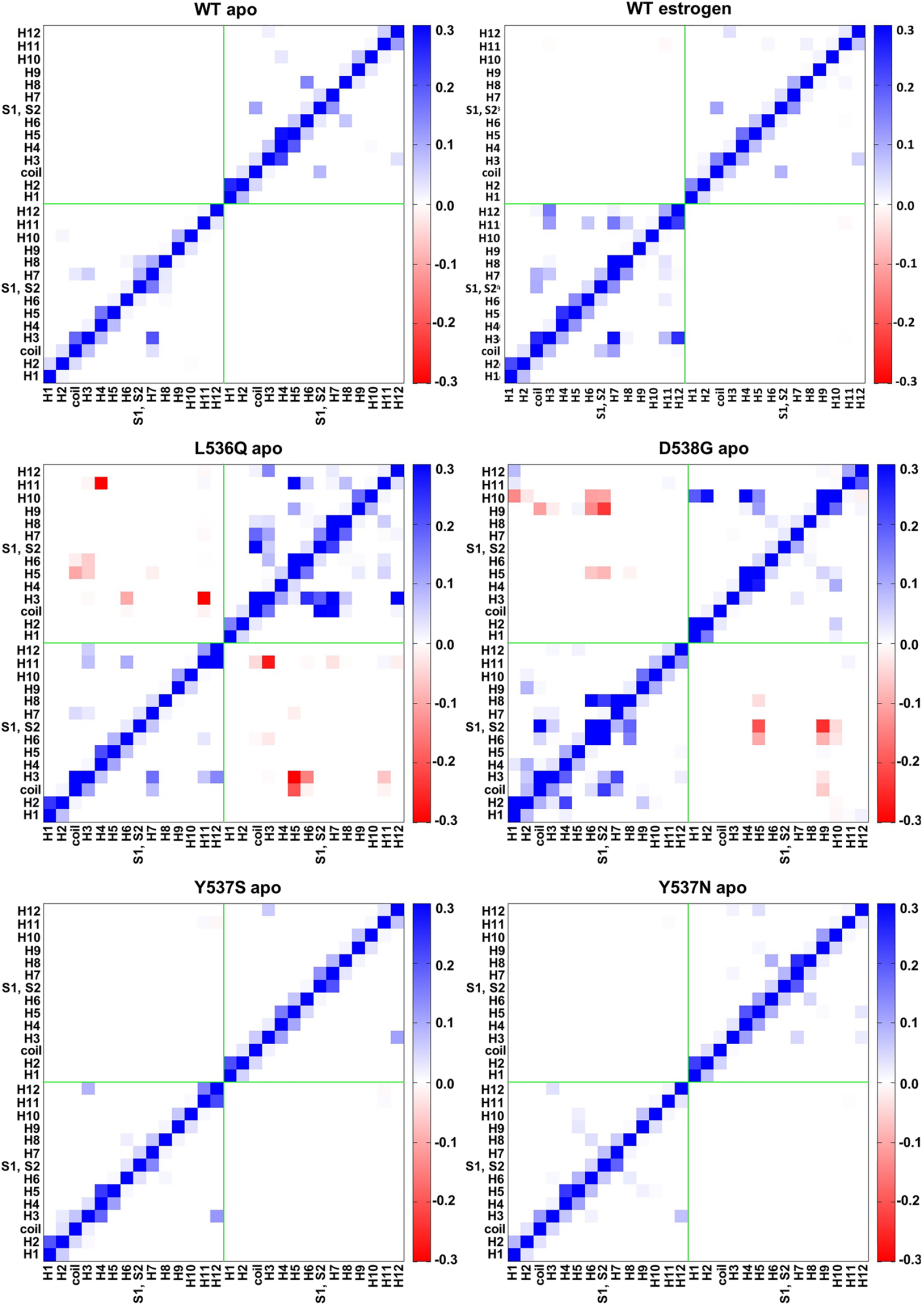
Cross-correlation maps of 14 regions (as defined in Fig. 1) of both, *apo* monomers of WT and the selected mERαs, and WT/EST. The cross-correlation coefficients, calculated as the sum of the cross-correlation coefficients (with a correlation score ≥ or ≤ than 0.6 and −0.6) of the residues belonging to two regions considered (see Paragraph S2 for details). Cross-correlation scores are reported in the range from −0.3 to 0.3 for clarity reasons. Blue and red colours account for positive and negative correlation, respectively.

Moreover, in the WT/EST a second contact is present between H7 and H3. This contact occurs only in the *apo* and EST bound forms of L536Q and D538G (hereafter L536Q/EST and D538G/EST), pinpointing a different activation mechanism with respect to the other two mutants. A clear anti-correlation between the two LBD monomers observed only in the *apo* L536Q and D538G (Figs 3 and S6) further confirms this. The presence of these mutants appears to reduce the symmetry of the two monomers, although we cannot exclude that this may be caused by a structural perturbation induced by the mutant, which is not recovered in our simulation time scale. Estrogen binding to mERαs reduces the observed anti-correlation between monomers and re-establishes the same correlation contacts of WT/EST (Fig. S6).

**Figure 4:**
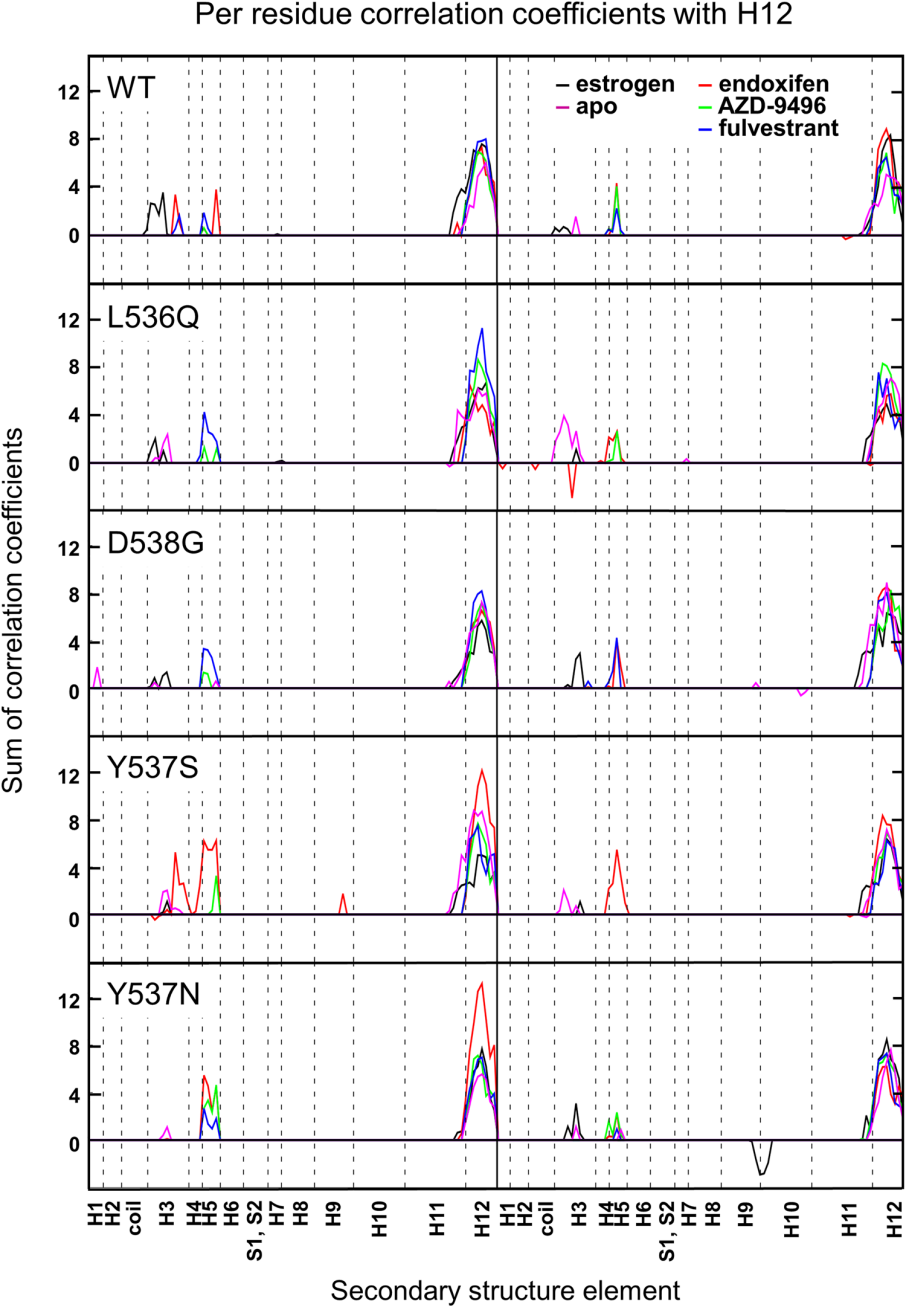
Sum of per-residue cross-correlation coefficients of H12 for the H12 residues with the rest of the LBD. Left and right columns refer to monomers A and B, respectively. From top to bottom: WT, L536Q, D538G, Y537S, and Y537N ERα isoforms are shown. Systems in the *apo* and estrogen-bound agonist forms are in magenta and black, respectively. Systems in the antagonist forms in complex with endoxifen, AZD-9496 and fulvestrant are shown as red, green, and blue lines, respectively.

Figure 4, displaying the correlation of H12 with the rest of the protein, gives a more semi-quantitative picture of the relative degree of activation of different isoforms, as emerging from our simulations. As such, we use the relative correlation score as a metric to discriminate between the ERα active and inactive states. Namely, the active EST/WT adduct has a correlation score of 4 between H12 and H3, which we take as a reference value for activation. Importantly, this plot enlightens that H12 correlates with H3 in all mutants, although with a lower score than the EST/WT adduct.

The second largest score, after EST/WT, is recorded for the *apo* L536Q and Y537S isoforms (both between 2 and 4), consistently with the fact that the latter is experimentally classified as the most aggressive variant. Conversely, the *apo* Y537N and D538G isoforms have a more limited intrinsic activation. From this graph it is also strikingly clear that all mutants are rather insensitive to the presence of EST.

Complementary analyses enlighten a mutant-dependent activation mechanism of ERα, with L536Q and D538G having a distinct activation procedure than Y537S and Y537N. Namely, the first two isoforms decrease the helical content of the H11/H12 segment, as previously suggested for D538G^22,28^ (Fig. S7), destabilizing the *apo* state. Instead, Y537S and Y537N induce the formation of hydrogen bonds (H-bonds) between H3 and L11-12 (*i*. *e*. O@L536···H_2_-Nδ2@N348, Oδ1/Oδ2@D351···H-N@D538, Oδ1/Oδ2@D351···H-N@L539, and Oδ1/Oδ2@D351···H-N@L540). Moreover, in Y537S there is an additional H-bond formed by the mutant side chain (Oδ1/Oδ2@D351···H-Oγ@S537), which further stabilizes the L11-12 (Fig. S8, Table S2).

### Antagonist conformation

#### Wild type ERα

We have considered here endoxifen (END) as traditional SERM, since this is the most abundant blood circulating metabolite of tamoxifen. When END binds to the WT ERα, monomers A and B display different correlation maps (Fig. S9), with H12 being weakly correlated with H3 and H5 in monomer A, and only with H5 in monomer B. These contacts, although weaker, are still present upon AZD-9496 and FULV binding. In all simulations, monomer A appears to be more sensitive than B to the presence of the antagonists, as shown by the analysis of the cross-correlation maps (Fig.s S9–13) (vide infra).

We exploit again the overall correlation of H12 with the rest of the protein as a metric of ERα activation. By this analysis emerges that when END binds to WT, H12 gains a correlation score of 4 with H3 and H5 in both monomers (Fig. 4). This may be consistent with the fact that END modulates the action of ERα, without completely inactivating it. Since, after many years of clinical use, it is clear that END beneficially antagonizes WT ERα, we take this correlation score as a threshold to assess whether a drug is effective/ineffective on the specific polymorphism investigated.

**Figure 5:**
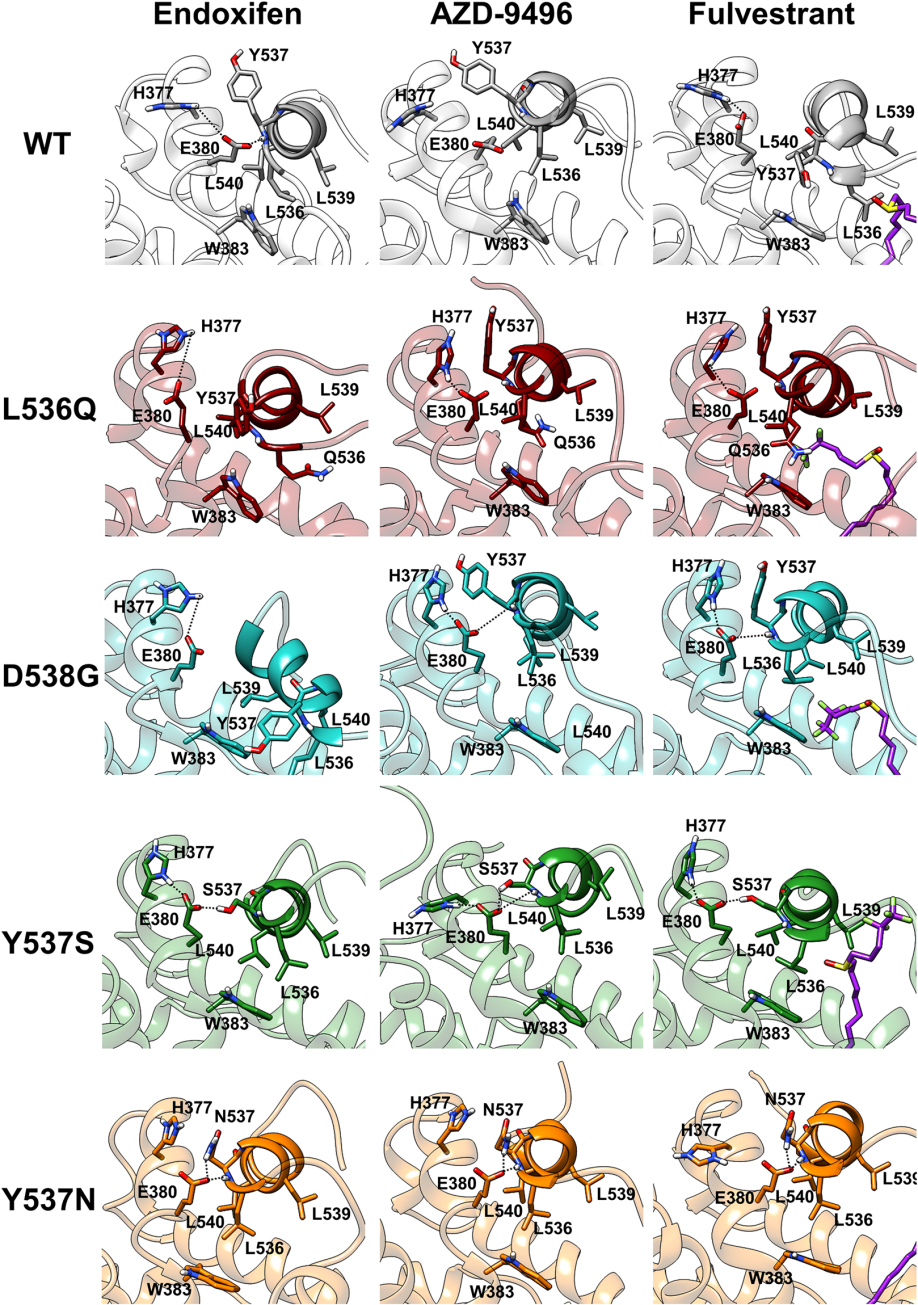
Interaction network of H12 with H5. In first, second, and third columns END-, AZD-9496-, and FULV-bound complexes are shown, respectively. WT (white) ERα and L536Q (dark red), D538G (turquoise), Y537S (green), and Y537N (orange) mERαs are shown from the top to the bottom row. FULV in the third column is coloured in violet. H-bonds are shown as black dotted lines. Oxygen, nitrogen, and hydrogen atoms are coloured in red, blue, and white, respectively. The structures depict the highest populated clusters of monomer A, as obtained from the cluster analysis (Table S1).

By comparing WT in complex with the different antagonists, one can see that in END/WT E380 H-bonds to the nearby H377 and that Y537 establishes π-stacking interactions with H377 (Fig.s 5, S14 and S15). In contrast, the persistence of this H-bond is slightly reduced and lost in the presence of FULV and AZD-9496 (Fig. 5, Table S3), respectively. This structural feature is, however, replaced by direct H-bonds between AZD-9496@O···H_3_Nζ@K529 and L346@O···H-N@AZD9496 (Fig.s S14, Table S4 and S5). FULV, instead, H-bonds with the ligand-binding pocket, similarly to END, and additionally establishes hydrophobic interactions with L11-12 (Tables S4 and S5, Fig. S14). Indeed, when FULV binds to WT ERα its long tail penetrates in the hydrophobic pocket formed by L536, W383, L539, and L540, interacting with L536 (Figs 5 and S13).

#### The L356Q Isoform

In the END and FULV/L356Q adducts H12 still correlates with H5 in one monomer, while this correlation almost vanishes upon AZD-9496-binding (Fig. S10). As mentioned above, the main effect of this isoform is mostly to induce disorder in H11, H12, and L11-12, by decreasing the helical content. This is confirmed by a decrease of the backbone H-bonds persistence between L536 and L540 in the presence of END, while both AZD-9496 and FULV partially recover them (Table S3).

Since the H-bond between the E380 and H377 side chains stabilizes the ERα-antagonist conformation, we take its absence as a signature of ERα (partial) activation. Consistently with this hypothesis, the E380Y mutation was among the first aggressive ERα variants identified, highlighting the importance of this H-bond for proper receptor function. For this reason, in the following discussion, we will monitor the presence/absence of the H-bond between E380 and H377 as a structural trait to rationalize the efficacy profiles of the drugs computationally assayed here. In L536Q/END this H-bond is present in both monomers, with its persistence even increasing in the presence of AZD-9406 and FULV (Fig. 5, Table S3).

In all L536Q ERα adducts the hydrophobic packing around residue 536 observed in the WT is disrupted by the introduction of the polar glutamine side chain. As a result, the secondary structure of H12 is distorted, and the π-π interaction between Y537 and H377 is lost in the END-bound complex, while FULV- and AZD-9496 partially and fully conserve it, respectively (Fig. S15). Again, in this polymorphism FULV penetrates deeply inside the hydrophobic pocket and establishes the largest non-bonded interactions with L11-12 and H12 (Table S6 and Fig. S14). Additionally, from the clustering analysis (Table S1) one can see that the antagonist conformation of the receptor is stabilized/rigidified the most by AZD-9496 followed by FULV. In constrast, a lower stability/larger flexibility of the structure is observed in the presence of END, where the highest populated cluster accounts for only 57% and 70% of all structures in monomer A and B, respectively.

#### D538G Isoform

Intriguingly, when END and AZD-9496 bind to D538G, a weak H12-H5 correlation is visible in monomer B, while upon FULV binding this becomes stronger in both monomers, but still below the aggressive activation threshold (Fig. S11). E380 H-bonds with H377, with a reduced persistence in the END-bound complex and with a conserved or increased persistence, with respect to the reference antagonist WT/END form, in the presence of FULV and AZD-9496 (Fig. 5, Table S3). FULV’s tail establishes again hydrophobic interactions with L536 (Fig. S14).

Remarkably, in the crystal structures of the agonist EST/WT form, a fragment of the coactivator protein was crystallized in the groove between H3 and H5 (PDB ids: 3uud^21^, 4zn9^27^, and 5jmm). In this structure, E380 forms a salt bridge with K688 of the coactivator fragment. In the antagonist WT/END form, this groove is occupied by H12, while E380 H-bonds to H377, preventing the binding of a coactivator protein.

Here we find evidences that in END/D538G H12 assumes a partial agonist conformation, forming a cavity within the H3–H5 groove, which may allow the binding of the coactivator protein (Fig. S16). This important finding further explains the reduced sensitivity of D538G to END and remarks the importance of E380 as on/off switch element of ERα mediated signal transduction.

The only available crystal structure of the receptor in the antagonist conformation and with one of the mutations studied is that of D538G mutant, where 4-hydroxy-tamoxifen is present in the binding site (PDB id: 4q50^22^). This structure, is, however, missing the L11-12 segment, hampering a detailed comparison with our results. The overall rigidity/stability of the receptor in the presence of all antagonists is high (over 95% of all structures, Table S1)

#### The Y537S Isoform

The correlation contacts of both monomers become very intense in Y537S/END (Fig. S12), while their intensity decreases and completely vanishes for Y537S/AZD-9496 and FULV, respectively. This is consistent with the proved efficacy of both drugs^18^. Upon END binding H12 displays the largest degree of correlation with H3 and H5 in monomer A and H5 in monomer B (Fig. 4), suggesting that END can only poorly counteract the intrinsic activation of the receptor. FUL and AZD-9496 eliminate this correlation contact, restoring a stable antagonist state.

The high degree of correlation between H12 and H5 observed for END/Y537S is accounted by the persistent H-bonds between E380 and both, the side chain and the backbone of S537. Their persistence is reduced by AZD-9496 (Table S3), which forms an extended network of H-bonds with the residues of L11-12, including S537 (Tables S4 and S5). Besides establishing hydrophobic interactions with the L536 pocket, FULV also H-bonds with the residues at the edge of H12 (533–536), resulting in reduced E380 H-bonding with the S537 side chain, thus decreasing the formation of this activation structural trait in both monomers (Table S3, Fig. 5 and S14).

The cluster analysis (Table S1) reveals that AZD-9496 is the SERD stabilize/rigidify the antagonist conformation the most in both monomers, followed by END and FULV, which both stabilize/rigidify the structure only in monomer B.

#### The Y537N Isoform

Y357N, the second most aggressive mutant among those investigated here, induces H12-H5 correlation contact only for monomer A in the presence of all considered drugs (Fig. S13), however, the correlation score remains at the edge of the selected aggressive activation threshold (Fig. 4). Only FULV reduces the correlation score below 4 in both monomers. In this mutant, the persistence of the H-bond between E380 and H377 is very low, while that between E380 side chain and N537 backbone and side chain is high. This structural signature is invariant even in the presence of drugs (Table S3): AZD-9496 forms a H-bond network up to residue 532 (Tables S4 and S5), while the interaction network of FULV is slightly more extended and complemented by hydrophobic interactions with L536.

Similarly, to Y537S, AZD-9496 is again the SERD stabilizing the most the antagonist conformation in both monomers (Table S1).

#### Ligand-receptor interactions: Implications for drug-design

All ligands occupy the active site in a similar manner, namely their heterocyclic rings have an orientation similar to that of EST. However, having distinct sizes, their tails interact differently with the receptor, especially in the L11-12 segment. In order to identify the subtle structural determinants allowing to rationalize the drug efficacy/inefficacy profile observed experimentally^18^, we have analysed the H-bonds and the hydrophobic interactions between the ligands and ERα as well as ligands’ stability within the binding pocket (Fig. S14).

Besides the interactions that the ligands establish with the binding pocket, AZD-9496 and FULV H-bond with the residues located in L11-12 (*i*. *e*. a salt bridge between carboxylic group of the ligand and K529 side chain in both monomers of WT, Y537S, and D538G in one monomer of L536Q), with the highest persistence being observed for D538G. This prevents H12 to interact with H5 and, in turn, to assume an agonist-like conformation.

In Y537S, the carboxylic group of AZD-9496 H-bonds with L536, C530, and with both, S537 backbone and side chain (Table S4), rationalizing AZD-9496 efficacy on Y537S. FULV, instead, persistently H-bonds with the backbone of L536. In all other isoforms, this H-bond is prevented, and FULV is mainly stabilized by hydrophobic interactions (Fig. S14), as shown by non-bonded interaction energies between the drug and the L11-12 and H12 (Table S6).

In summary, AZD-9496 and FULV, due to their elongated shape, can form either H-bonds (mostly with K529 and K531) or hydrophobic interactions with residues of L11-12 and H12, hampering H12 from establishing a correlation contact with H5, and reducing the degree of ERα activation. As such, the direct interaction of the ligand with the L11-12 and, in particular, with the residues in the region 529–537, is crucial to exert an antagonistic/downregulator activity even in aggressive polymorphisms. This appears to be in line with recently identified ERα downregulators, which bear a carboxyl moiety pointing towards L11-12^29^ and with the hydrophobic interactions with L536 observed for a potent antagonist with a new scaffold^30^.

In consequence, we expect that all drugs exhibiting efficacy against these aggressive mutants should carry H-bond-forming moieties up to the region 529–537, thus hampering the selection of an agonist-like conformational state in these polymorphisms (Fig. S17).

### Summary and Conclusions

Understanding at atomistic level of detail how aggressive polymorphic variants of ERα trigger intrinsic ERα activation, along with the structural traits responsible for the improved efficacy profile of clinically used SERDs against these variants, is a prerequisite for personalized therapies to treat metastatic BC patients relapsing fist-line endocrine treatments.

In this study, we performed cumulative 12.5 µs (*i*. *e*. 500 ns for each investigated system) classical MD simulations to picture the complex conformational landscape of recurrent and aggressive pro-oncogenic mutations showing that: (i) all investigated *apo* ERα isoforms (L536Q, Y537S, Y537N, D538G), are constitutively active, explaining why these variants are refractory to therapies based on HA inhibitors. Nevertheless, our study strikingly reveals that each isoform possesses a unique activation mechanism. Namely, when the polymorphism occurs at position 537, direct H-bonds are engaged between H12 and N348 and/or D351, making these mutants the most aggressive. In contrast, in L536Q and D538G, the mutations induce an overall disorder in the secondary structure at the edge of H12 and a decrease of its helical content; (ii) When introducing the polymorphic variants in the ERα antagonist state, a shift toward an agonist-like conformation occurs due to a structural rearrangement of L11-12 and to the formation of H-bonds between the mutated residues belonging to H12 and E380.

This structural transition is crucial for ERα activation, even in the presence of END, and it is partially hampered by the two SERDs computationally assayed here. Our study remarks the importance of E380 to regulate ERα activation mechanism, as confirmed by genomic studies^31^. (iii) The efficacy of AZD-9496 toward all ERα isoforms investigated may be attributed to a specific set of H-bonds that this drug establishes with the residues of the L11-12, mainly with K529/531. This H-bond network prevents the conformational transition of H12 towards the agonist-like state; (iv) The long FULV hydrophobic tail establishes hydrophobic interactions with L11-12 and H12 (Table S6), which again impedes the shift towards an agonist-like ERα active conformation. (v) Finally, D538G/END induces the conformational selection of a H12 state, creating a cavity able to bind a co-activator peptide. This suggests a potential alternative mechanism of counteracting the activity of this mutant.

As a note of warning, we remark that the length of our MD simulations is sufficient to explore the potential energy surface nearby the starting configuration. Large conformational changes, occurring on multiple microseconds/millisecond time-scale induced by the mutants or the drugs, will not be fully captured here. Since ESR1 mutations in metastatic BCs are highly recurrent, a detailed atomistic understanding of the structural traits needed by efficacious drugs can lead research efforts to identify more effective ERα antagonists/downregulators. Furthermore, our computational assay, providing for the first time a rational understanding of efficacy of drugs in (pre-)clinical use for each ERα isoform, can lead physicians to devise optimal personally tuned therapies on the basis of patients’ genetic profile.

### Computational Methods

#### ERα models

The WT model was built starting from the crystal structure of 17-β-estradiol (EST)-bound ERα dimer (PDB id: 1qku^26^). The *apo* form model was generated by removing EST from the binding site.

The antagonist adducts between END/AZD-9496 and WT ERα were built from the crystal structures of ERα monomer in complex with 4-hydroxy-tamoxifen and AZD-9496 (PDB ids: 3ert^32^ and 5acc^19^, respectively). To generate END from the crystal structure, we deleted one of the two terminal methylene groups of 4-hydroxy-tamoxifen. Then, we built the LDB dimer in the antagonist state by superimposing monomeric antagonist ERαs structures, solved by X-ray crystallography (PDB ids: 3ert^32^ or 5acc^19^), on the both monomers of the antagonist ERα crystal structure (PDB id: 1qku^26^). Since the agonist and antagonist structures differ only in the orientation of the H12, we expect the overall folding of the LDB monomers to be quite similar. Nonetheless, we verified the stability of the built dimers during the MD simulations (Fig. S3). The residues missing in the crystal structure of AZD-9496/ERα adduct (PBD id: 5acc^19^) were modelled according to the crystal structure of 4-hydroxy-tamoxifen-bound ERα.

The crystal structure of FULV-bound ERα has not been solved, thus we built this model by removing 4-hydroxy-tamoxifen from the crystal structure of antagonist ERα, and docking FULV in the binding site with the Glide software^33,34^. mERα models of all the antagonists and agonists adducts were built by making point mutations L536Q, Y537S, Y537N and D538G on the models with the Rotamers tool of UCSF Chimera^35^. This is based on the Dunbrack backbone-dependent rotamer library^36^. In total, we constructed 25 systems. Protonation states of ionisable residues have been determined according to H++ server^37^.

#### Molecular Dynamics (MD) Simulations

Each model was solvated with TIP3P water molecules in the truncated octahedron shaped box with the dimensions of at least 14 Å in each direction from the solute. In all simulations we used Amber99SB force field for protein^38^. Na^+^ ions were added to achieve system neutrality. Systems of agonist and antagonist adducts are composed by 81000 and 106000 atoms, respectively. The partial charges of each ligand were obtained by performing population analysis according to the Merz-Kollman scheme on the geometry optimized structures at Hartree-Fock level with 6–31 G* basis set with the Gaussian09^39^. RESP charges were the generated with the Antechamber module of Amber13^40^. The other ligands force field parameters were obtained with Antechamber module, using as input bond lengths and bond angles obtained from the optimized geometries.

Prior to the production MD simulations, all systems were heated to the final temperature of 310 K using 40 steps of simulated annealing (0–90 K in steps of 5 K/25 ps; 90–310 K in steps of 10 K/25 ps). Production runs were done with periodic boundary conditions at 310 K and 1 bar by coupling to the Nose-Hoover thermostat^41,42^ and Parrinello-Rahman barostat^43^. Electrostatic interactions were taken into account using particle mesh Ewald method^44^. A time step of 2 fs was used, constraining bond lengths with the LINCS algorithm^45^. 500 ns long simulations were performed for each system and last 300 ns were considered for further analysis. Simulations were performed using Gromacs5.1.2 code^46^.

#### Analysis

The root mean square displacement (RMSD) and root mean square fluctuation (RMSF) were calculated with gmx_rms and gmx_rmsf modules of Gromacs 5.1.2, respectively. Trajectories were clustered using gmx_cluster module of Gromacs 5.1.2 according to their RMSD value using the clustering method reported by Daura *et al*.^47^. The selected RMSD cut-off value for clustering analysis was 2.75 Å.

The *cpptraj* module of Amber program was used for hydrogen bond analysis (cut-off parameters for H-bond was 3.3 Å and 35°), cross-correlation matrices, and analysis of the secondary structure^48^. For details on cross-correlation matrices calculations see Paragraph S2.

## Electronic supplementary material

Supplementary Information
