# Supplementary material for: A Computational Assay of Estrogen Receptor α Antagonists Reveals the Key Common Structural Traits of Drugs Effectively Fighting Refractory Breast Cancers
Source: Sci Rep. 2018 Jan 12;8:649. doi: 10.1038/s41598-017-17364-4 (PMC5766519; doi:10.1038/s41598-017-17364-4)
Supplement: Supplementary file 1 — Supplementary Information [file 41598_2017_17364_MOESM1_ESM.pdf]

Supporting Information

A Computational Assay of Estrogen Receptor α Antagonists Reveal the Key Common Structural Traits of Drugs Effectively Fighting Refractory Breast Cancers

Matic Pavlin,[a] Angelo Spinello,[a] Marzia Pennati,[b] Nadia Zaffaroni,[b] Silvia Gobbi,[c] Alessandra Bisi,[c] Giorgio Colombo[d] and Alessandra Magistrato[*,a]

a CNR-IOM-Democritos c/o International School for Advanced Studies (SISSA), via Bonomea 265, 34136 Trieste, Italy

b Fondazione IRCSS Istituto Nazionale dei Tumori, via Amadeo 42, 20113 Milano, Italy

c Department of Pharmacy and Biotechnology, Alma Mater Studioum-University of Bologna, Via Belmeloro 6, 40126 Bologna, Italy

d CNR-ICRM via Mario Bianco 9, 20131 Milano, Italy

Content

1. Supporting Text S1 and S2

2. Supporting Figures S1 to S17

3. Supporting Tables S1 to S6

Supporting Text

S1. RMSF of agonist and antagonist ERα States

The first peak is located between residues 328 and 343 corresponds to the coil between H2 and H3 (hereafter named Coil2-3), Fig. S4 shows that the flexibility of this region is independent from both the ERα polymorphisms and the ligand type present in the binding site. The second peak lies between residues 458 and 478. These residues are part of H10, which, however, is involved neither in ligand binding nor interacting with the H12. By comparing the flexibility of these regions for the different mutants it is clear that no large differences are present and that in the presence of estrogens, (17-beta-estradiol) the effect of the mutant is reduced with respect to the WT. The only region in which the *apo* form has an increased flexibility is in the tract comprising 523 to 533 residues, located on the coil connecting H11 and H12. This results to be slightly more flexible in the *apo* form of all polymorphic variants.

Remarkably, in addition to the regions characterized by high mobility we register for the agonist conformation, the residues of the coil connecting H11 and H12. The mutants induce overall larger flexibility with respect to the WT. It is evident that AZD-9496 and fulvestrant, which are markedly larger than endoxifen, perturb the flexibility of this region even in the WT. The only mutant having a rigid structure independently of the ligand bound is the D538G, although the fluctuations in this region in the presence of endoxifen are larger for the polymorphic variants than for the WT. It is strikingly clear that in all polymorphic variants of ERα (mERαs) this coil is more flexible than in the WT complex with endoxifen, probably due to its tendency to rearrange towards an agonist-like conformation, as observed in other biophysical studies.[1,2]

In the endoxifen bound complexes these fluctuations are the lowest in the case of WT ERα, whereas they are evident in both monomers in the L536Q and Y537S polymorphisms. This suggests that endoxifen stabilizes the coil between H11 and H12 only in WT ERα, whereas in the mERα mutations induce coil's increased flexibility. Since the mutations lies within this coil it is clear that their impact on the flexibility of the antagonist form is huge.

S2. Cross-correlation matrix calculations

Cross-correlation matrix – based on Pearson coefficients – between the fluctuations of the Cα atoms relative to their average positions has been calculated to establish how the motions between the protein residues are correlated. Positive values describe a correlated motion between atoms, while negative values refer to the anti-correlated motions.

To provide a clearer picture of the correlation motion the per-residue cross correlation coefficients have been computed considering the only the most positive (≥ 0.6) and negative (≤ –0.6) Pearson correlations.

Since here we are considering only the LDB ERα inter and intra structural element correlations have been calculated by considering the cross correlation score of the sum of each residues within a selected region with the residues belonging to the different or the same structural element of the residue in consideration. These account for the degree of correlated and anti-correlated motion displayed by each region with the rest of the system. This has been done according to the structural motifs introduced in Figure 1, *i. e.* by dividing each monomer in 14 regions (as shown in Fig. 1 of the main text).[3] The cross correlation maps are normalized by columns (i.e. the cross correlation score are normalized with respect to the region, which in each column has the largest sum of the cross-correlation scores). Thus, these maps shows how each structural element on the x-axis correlates with any other structural element reported y-axis. Cross-correlation scores are reported in the range from -0.3 to 0.3 for clarity reasons. Blue and red colours account for positive and negative correlation, respectively.

We also show how each All figures of (m)ERαs were generated with UCSF Chimera visualization tool.[4] Plots were generated with gnuplot and xmgrace.

2. Supporting Figures

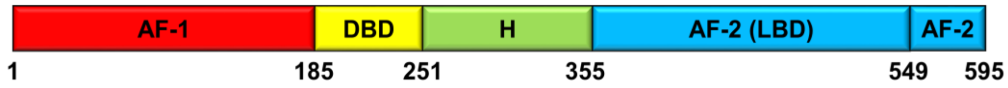

Figure S1. Schematic representation of five distinct functional domains of ERα: activation function domains AF-1 and AF-2, DNA-binding domain (DBD), hinge (H) and ligand-binding domain (LBD; which is part of AF-2). While LBD and DBD have ordered secondary structure, AF-1, non-LBD part of AF-2 and the hinge are structurally disordered. Thus, the tertiary structure of the full-length receptor is not known.

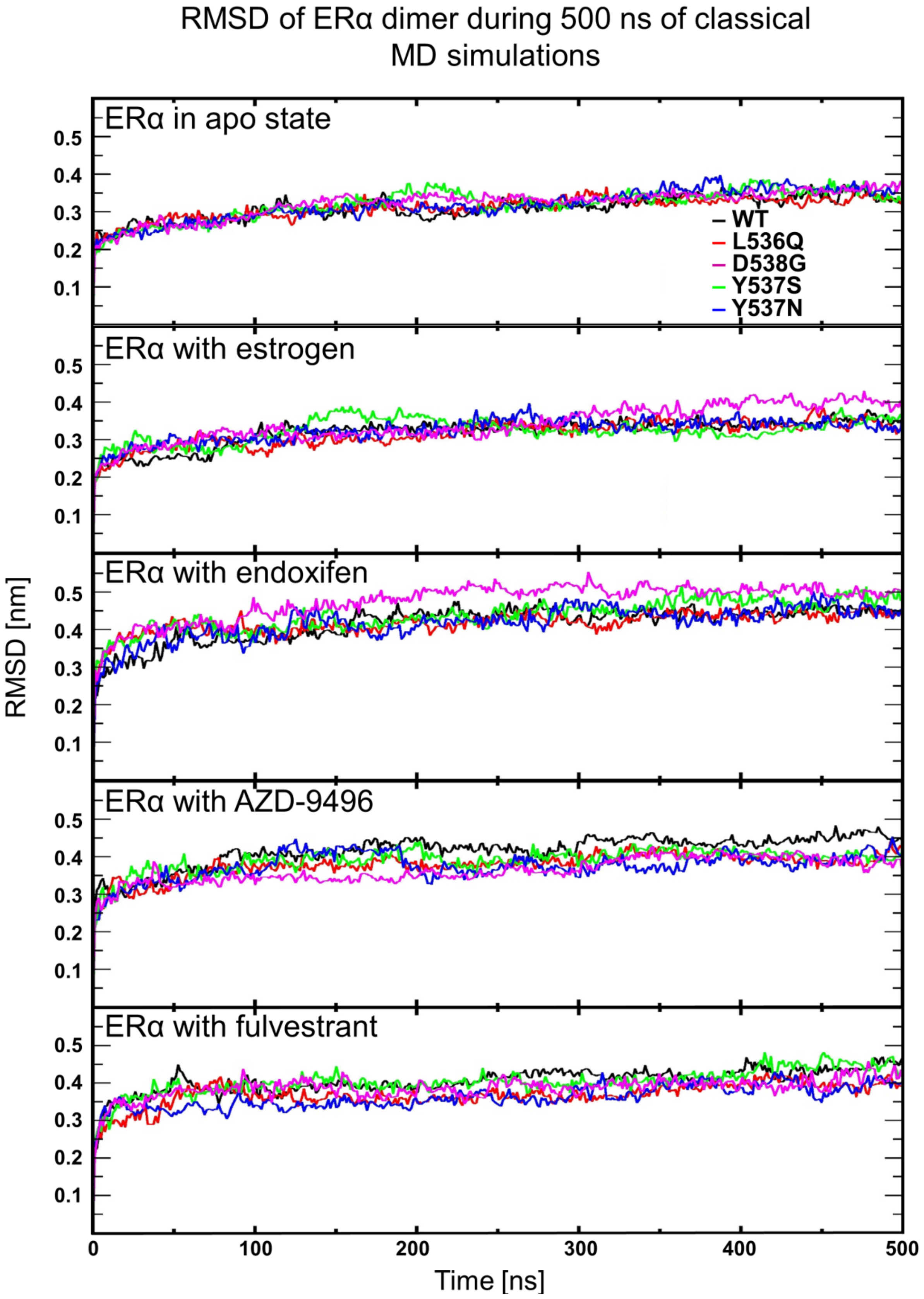

Figure S2. RMSD (nm) plots of ERα LBD dimer. 500 ns simulations of WT and mERαs in *apo* state and in complex with estrogen, endoxifen, AZD-9496 and fulvestrant (from top to bottom). WT ERα is plotted in black and mERα in red (L536Q), magenta (D538G), green (Y537S), and blue (Y537N).

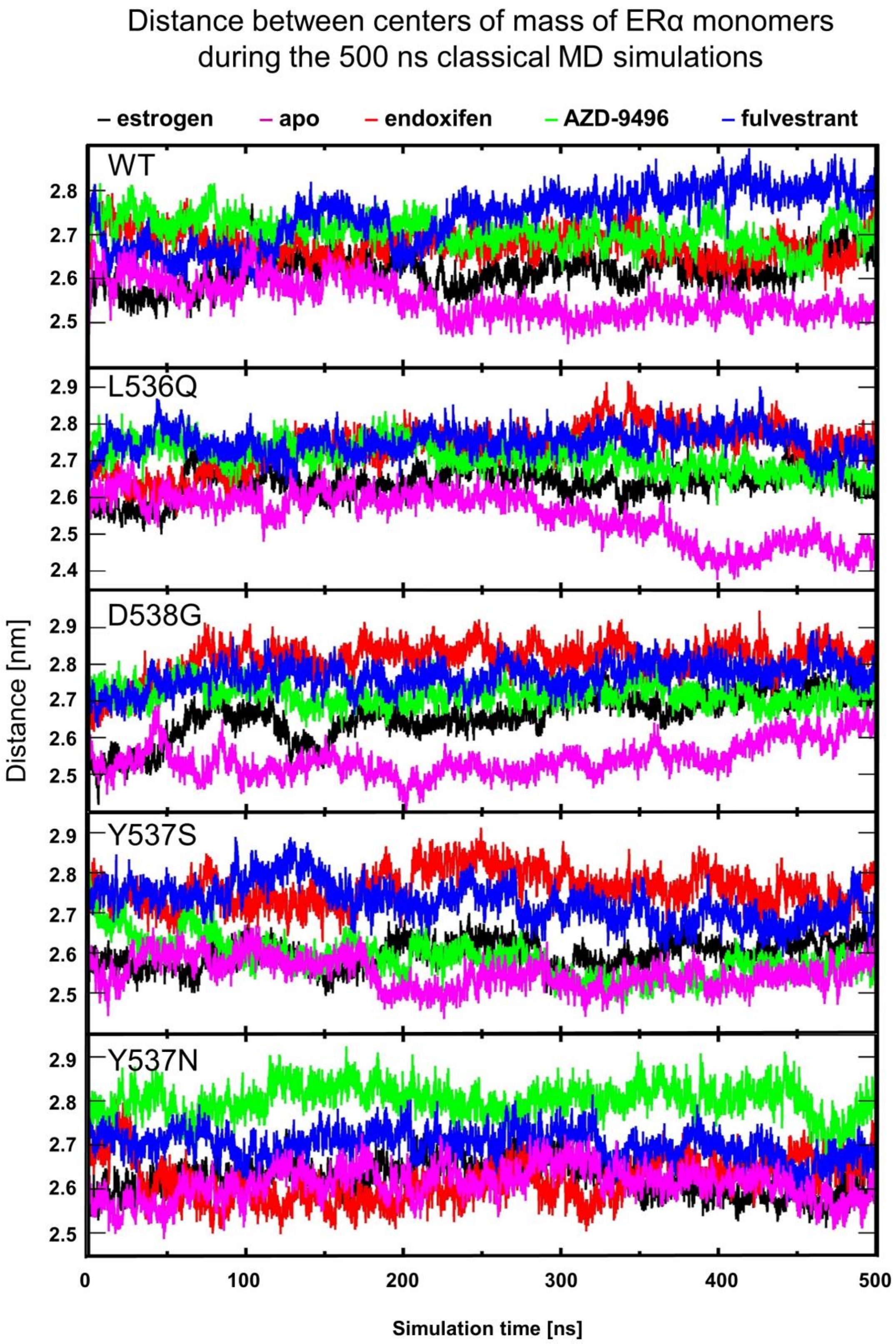

Figure S3. Distance (nm) between the centres of mass of monomers A and B of the LDB vs simulation time. WT ERα is plotted in black and mERαs are shown in red (L536Q), magenta (D538G), green (Y537S), and blue (Y537N).

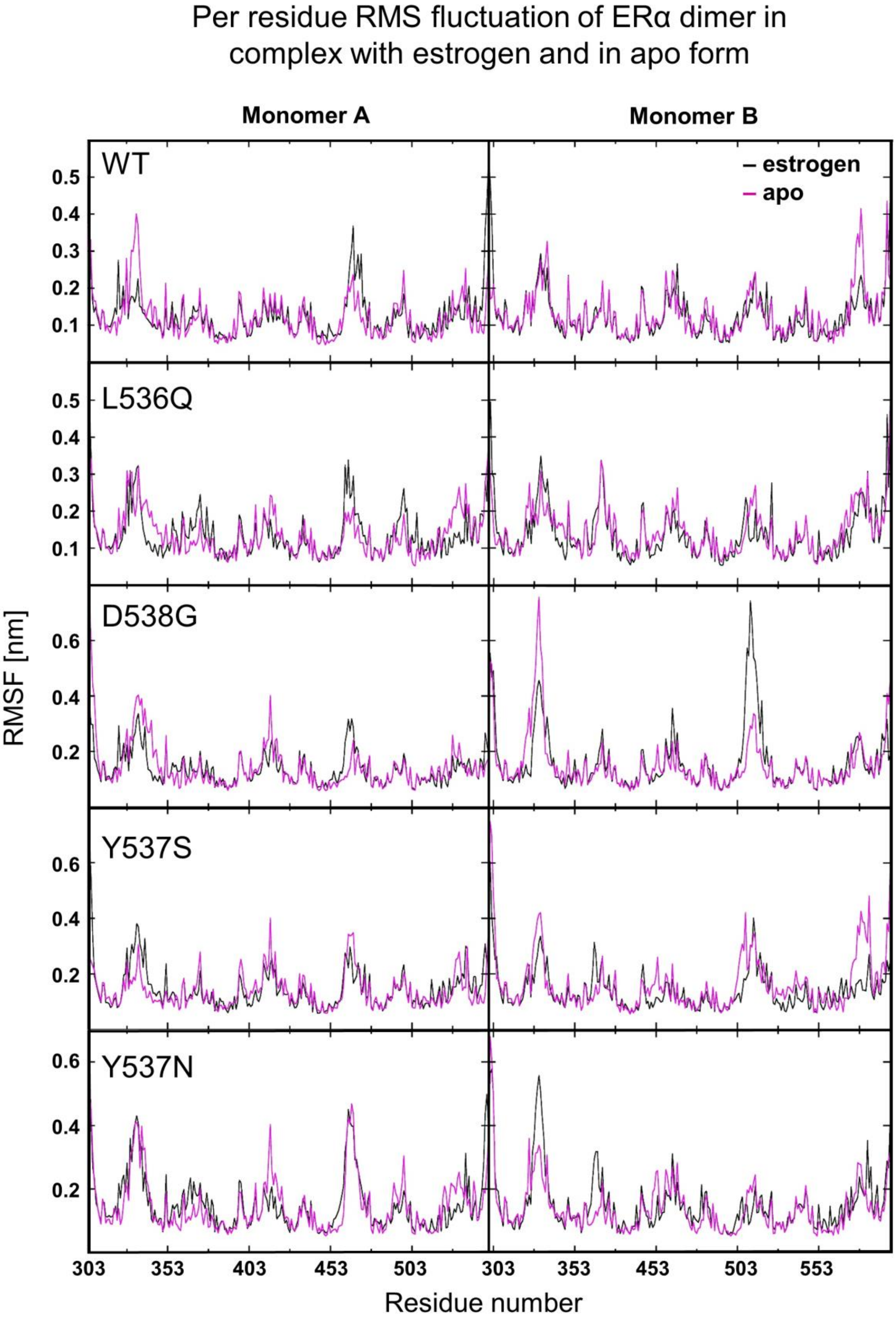

Figure S4. Per residue RMSF (nm) for the *apo* (magenta) and the estrogen-bound (black) form of the WT ERα and the four selected mERα polymorphisms (L536Q, D538G, Y537S, and Y537N), considering the receptor in the agonist state. Monomer A and B are shown on the left and right, respectively.

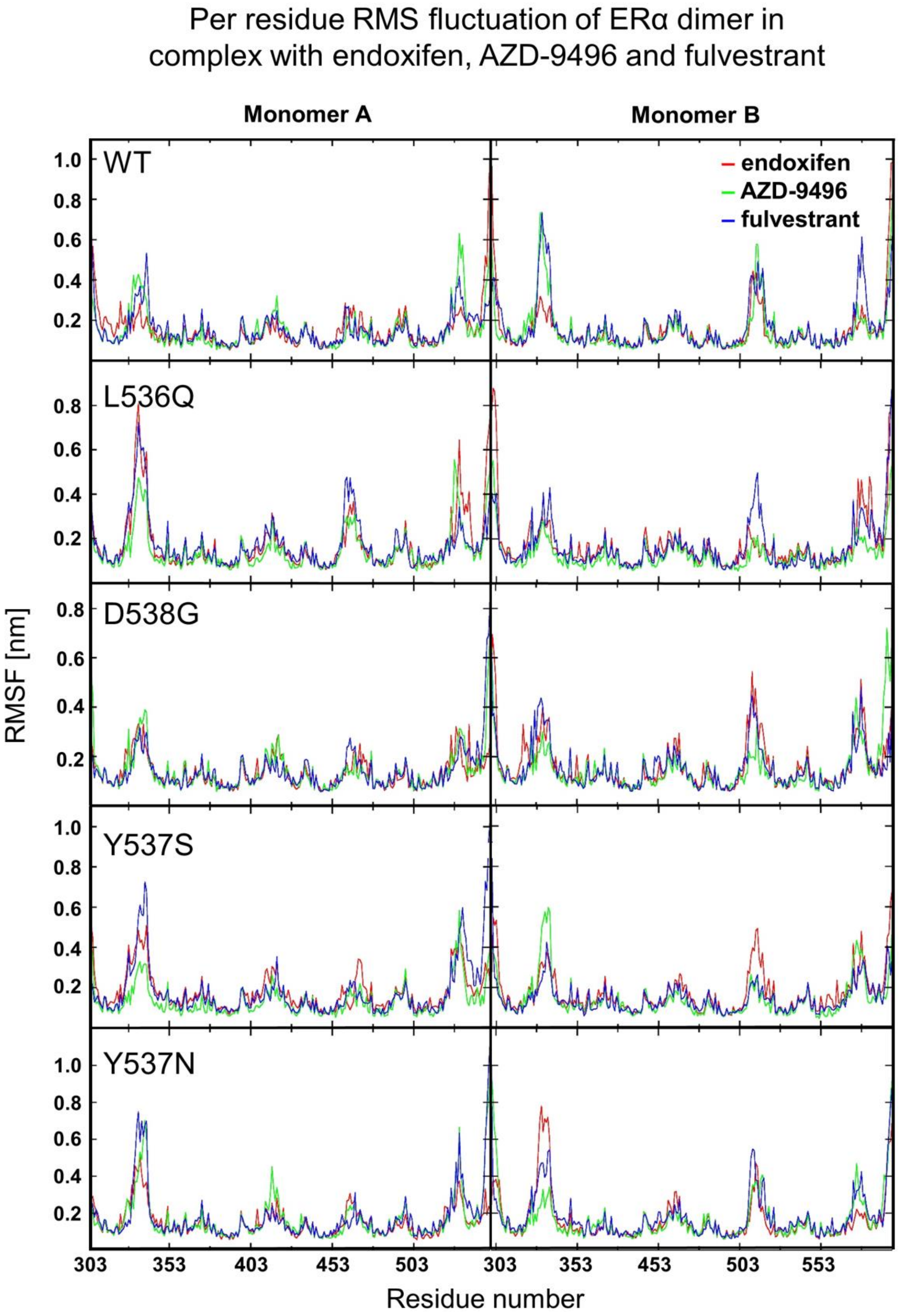

Figure S5. Per residue RMSF (nm) for the END-, AZD-9496-, and FULV-bound form of the WT ERα and the four selected mERα polymorphisms (L536G, Y537S, Y537N, D538G) from top to bottom, considering the receptor in the antagonist state. Monomer A and B are shown on the left and right, respectively.

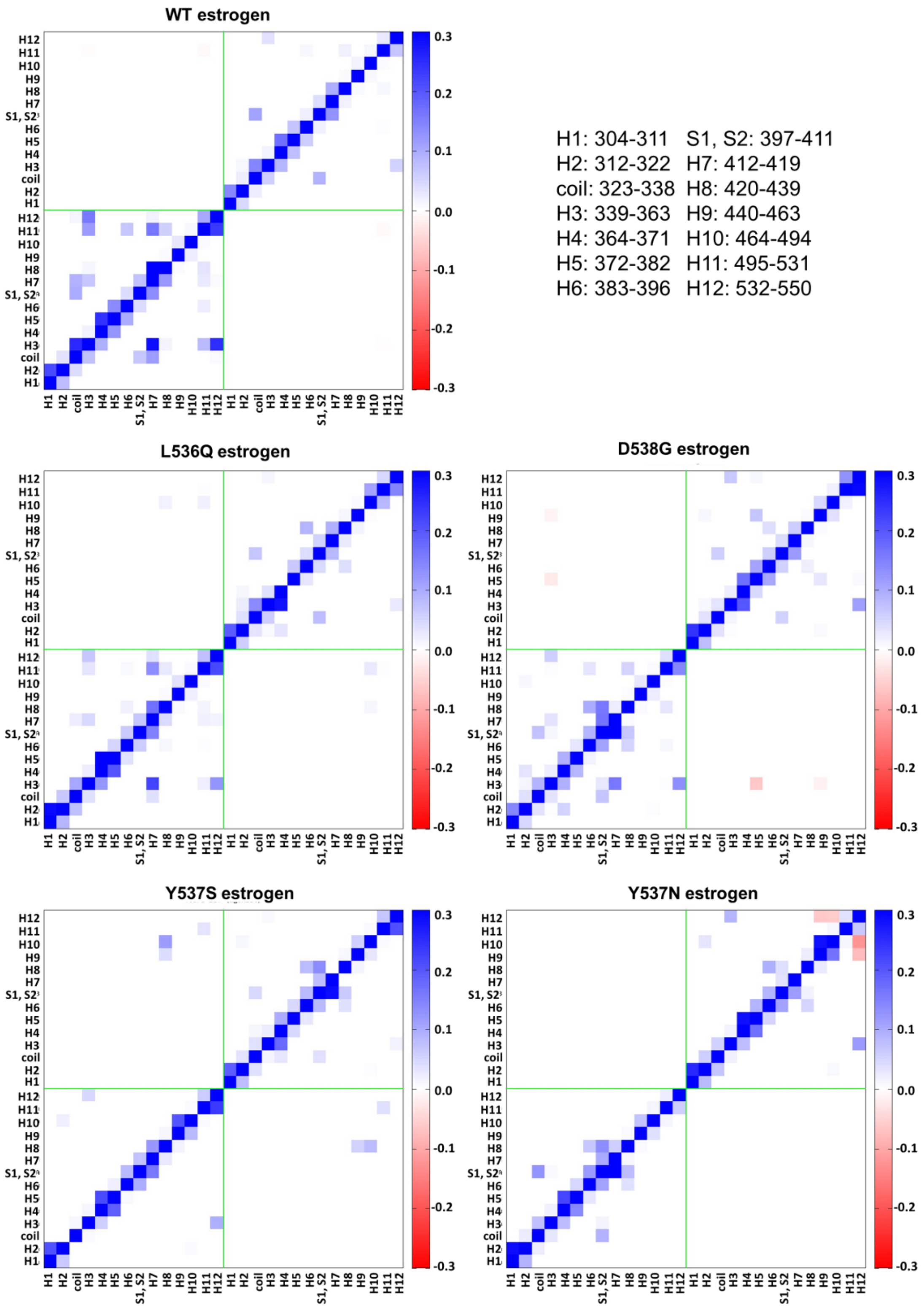

Figure S6. Cross-correlation maps of 14 regions (as defined in Fig. 1 of the main text) of both monomers of WT ERα and all mERαs in the *estrogen*-bound form. The cross-correlation coefficients, normalized by columns, are reported in the range from -0.3 to 0.3 due to clarity reasons. Blue and red colours account for positive and negative correlation. Top left is WT ERα, middle left L536Q ERα, middle right D538G ERα, bottom left Y537S ERα and bottom right Y537N ERα. Cross-correlation scores are reported in the range from -0.3 to 0.3 for clarity reasons. Blue and red colours account for positive and negative correlation, respectively.

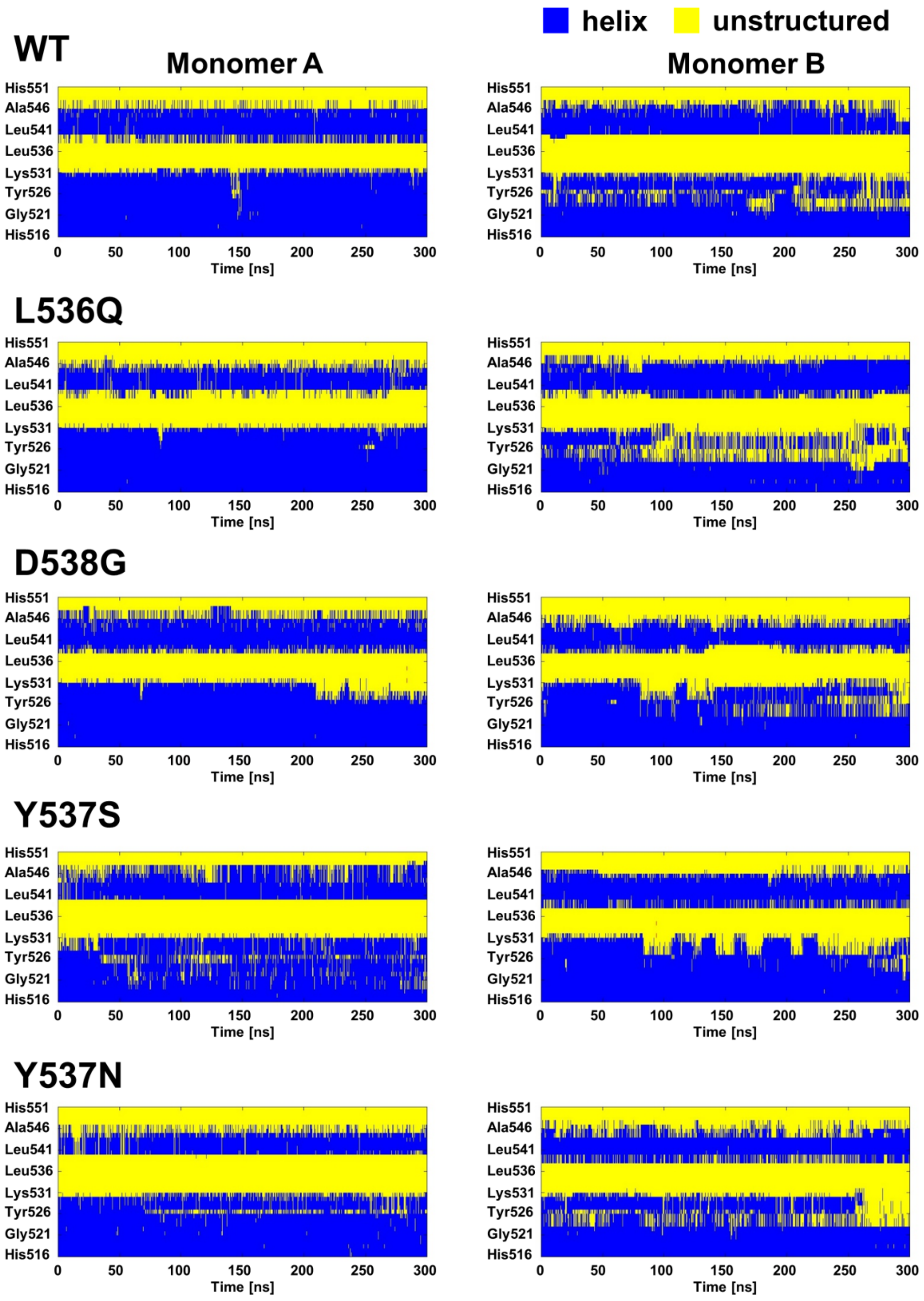

Figure S7. Secondary structure elements (colored in blue is helix and in yellow is unstructured part of the receptor) of the *apo* ERα (WT and all polymorphisms) for the last 35 residues of monomer A (left column) and B (right column), as calculated from the last 300 ns of MD simulations.

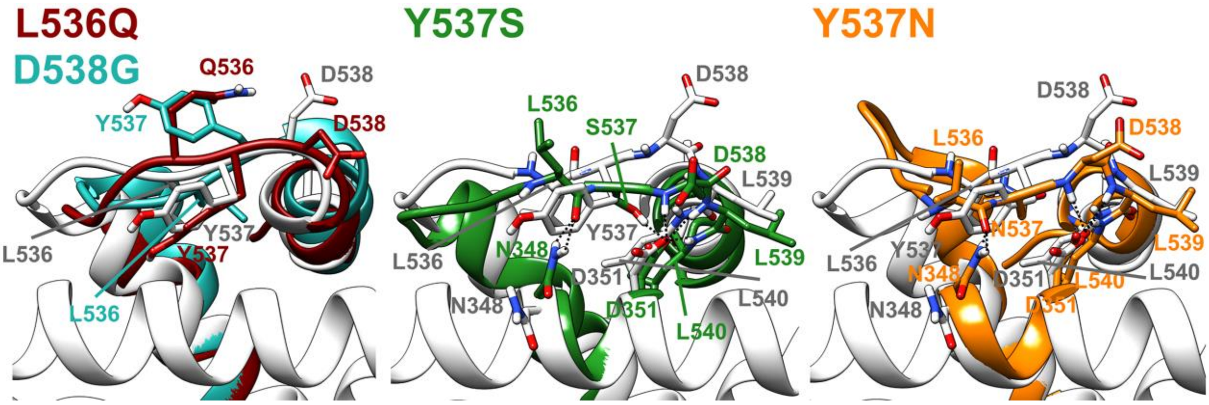

Figure S8. Interactions between the L11-12 and H3 for all *apo* mERαs (L536Q in dark red, D538G in turquoise, Y537S in green and Y537N in orange colour) in comparison with the WT ERα (white) in agonist state. Oxygen atoms are shown in red, nitrogen in blue and polar hydrogen atoms in white. Nonpolar hydrogen atoms are omitted for clarity. H-bonds are shown as dotted black lines. Structures of most populated cluster, obtained by the analyses of the last 300 ns simulations, for the ERα monomer A are shown.

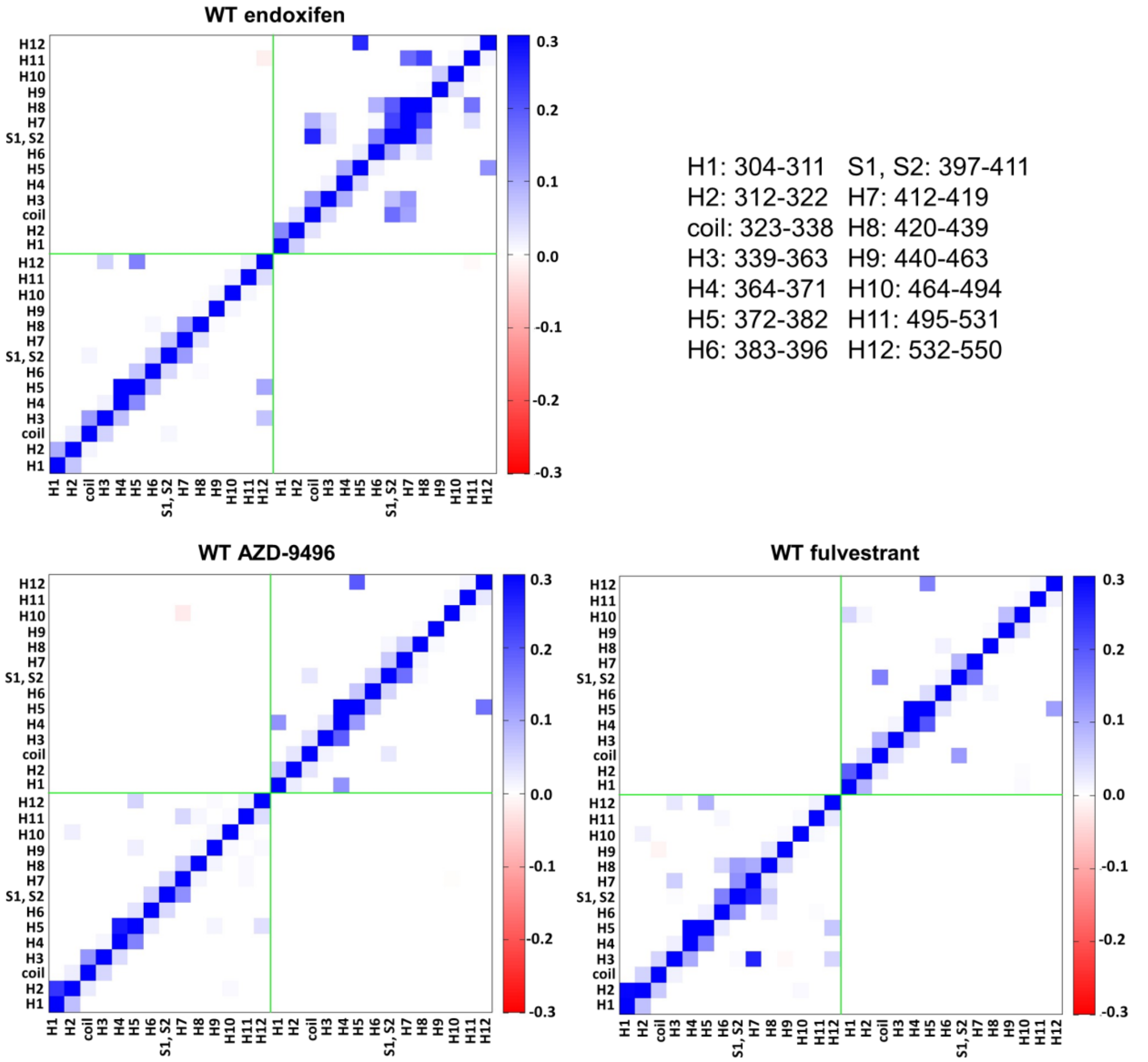

Figure S9. Per-column normalized cross-correlation maps of 14 regions (as defined in Fig. 1 of the main text) of both monomers of WT ERα in *antagonist*-bound form. The cross-correlation coefficients, normalized by columns, are reported in the range from -0.3 to 0.3 due to clarity reasons. Blue and red colours account for positive and negative correlation. Top left is END-bound ERα, bottom left AZD-9496-bound ERα, and bottom right FULV-bound ERα.

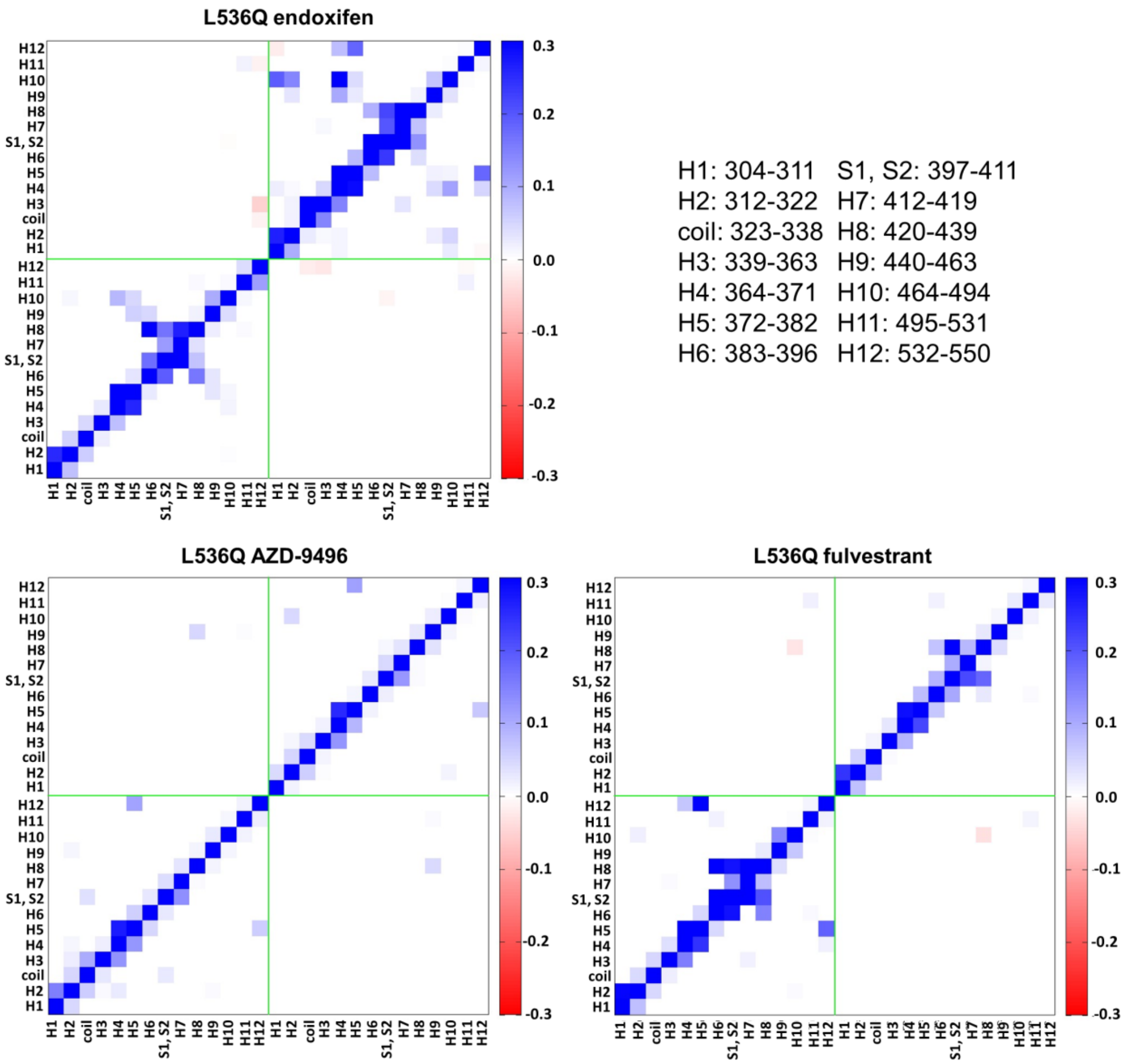

Figure S10. Per-column normalized cross-correlation maps of 14 regions (as defined in Fig. 1 of the main text) of both monomers of L536Q ERα in *antagonist*-bound form. The cross-correlation coefficients, normalized by columns, are reported in the range from -0.3 to 0.3 due to clarity reasons. Blue and red colours account for positive and negative correlation. Top left is END-bound ERα, bottom left AZD-9496-bound ERα, and bottom right FULV-bound ERα.

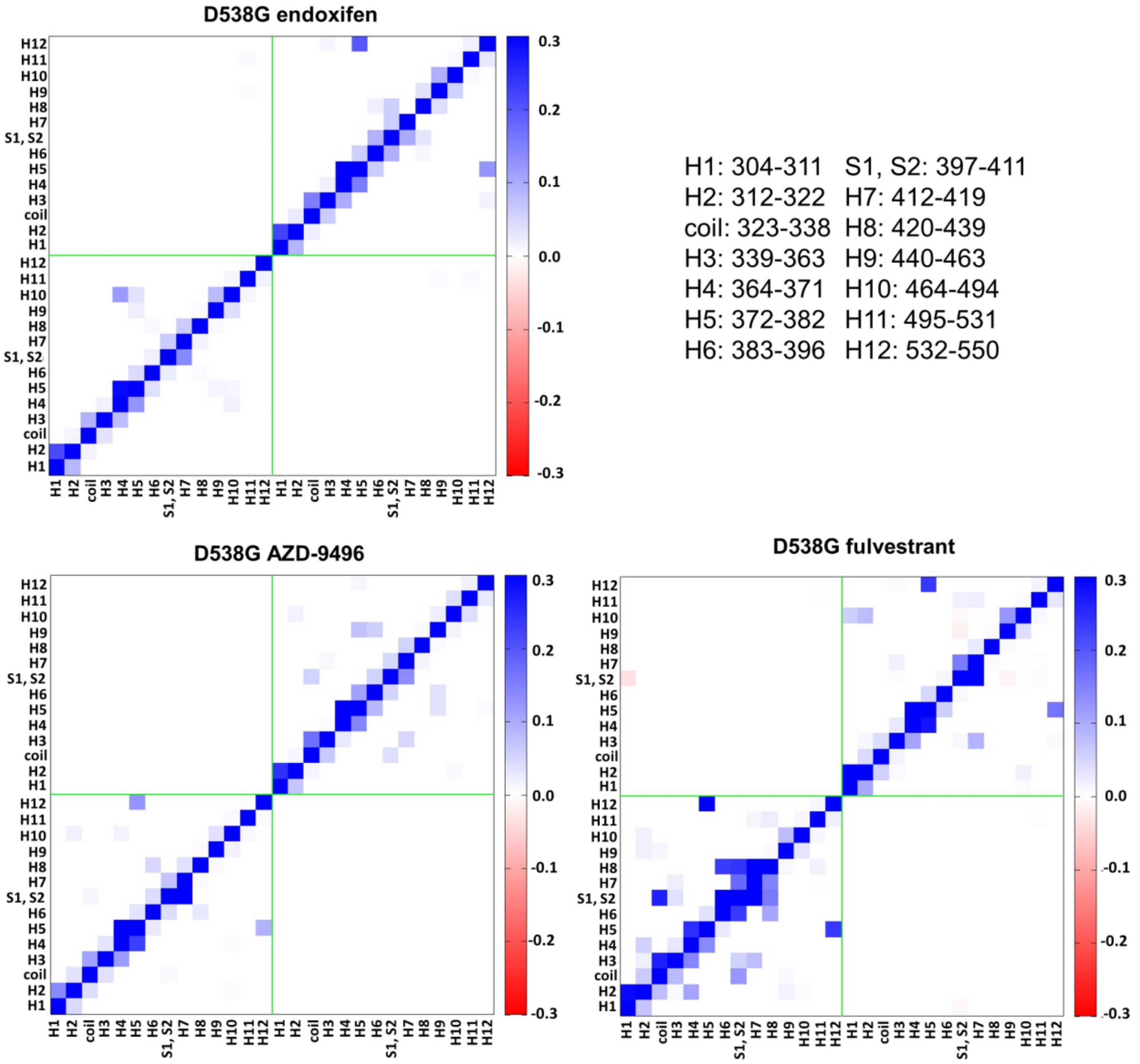

Figure S11. Per-column normalized cross-correlation maps of 14 regions (as defined in Fig. 1 of the main text) of both monomers of D538G ERα in *antagonist*-bound form. The cross-correlation coefficients, normalized by columns, are reported in the range from -0.3 to 0.3 due to clarity reasons. Blue and red colours account for positive and negative correlation. Top left is END-bound ERα, bottom left AZD-9496-bound ERα, and bottom right FULV-bound ERα.

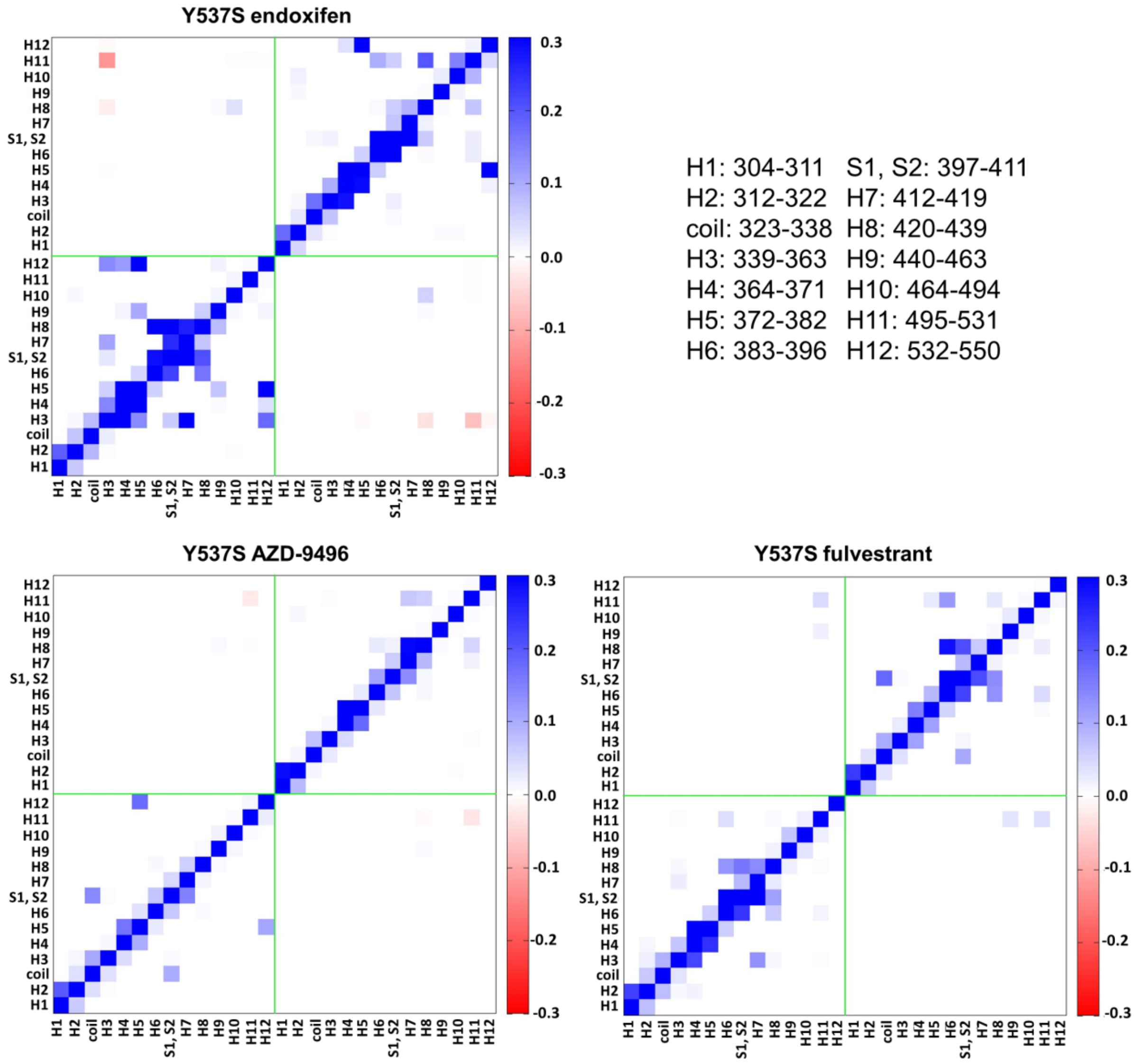

Figure S12. Per-column normalized cross-correlation maps of 14 regions (as defined in Fig. 1 of the main text) of both monomers of Y537S ERα in *antagonist*-bound form. The cross-correlation coefficients, normalized by columns, are reported in the range from -0.3 to 0.3 due to clarity reasons. Blue and red colours account for positive and negative correlation. Top left is END-bound ERα, bottom left AZD-9496-bound ERα, and bottom right FULV-bound ERα.

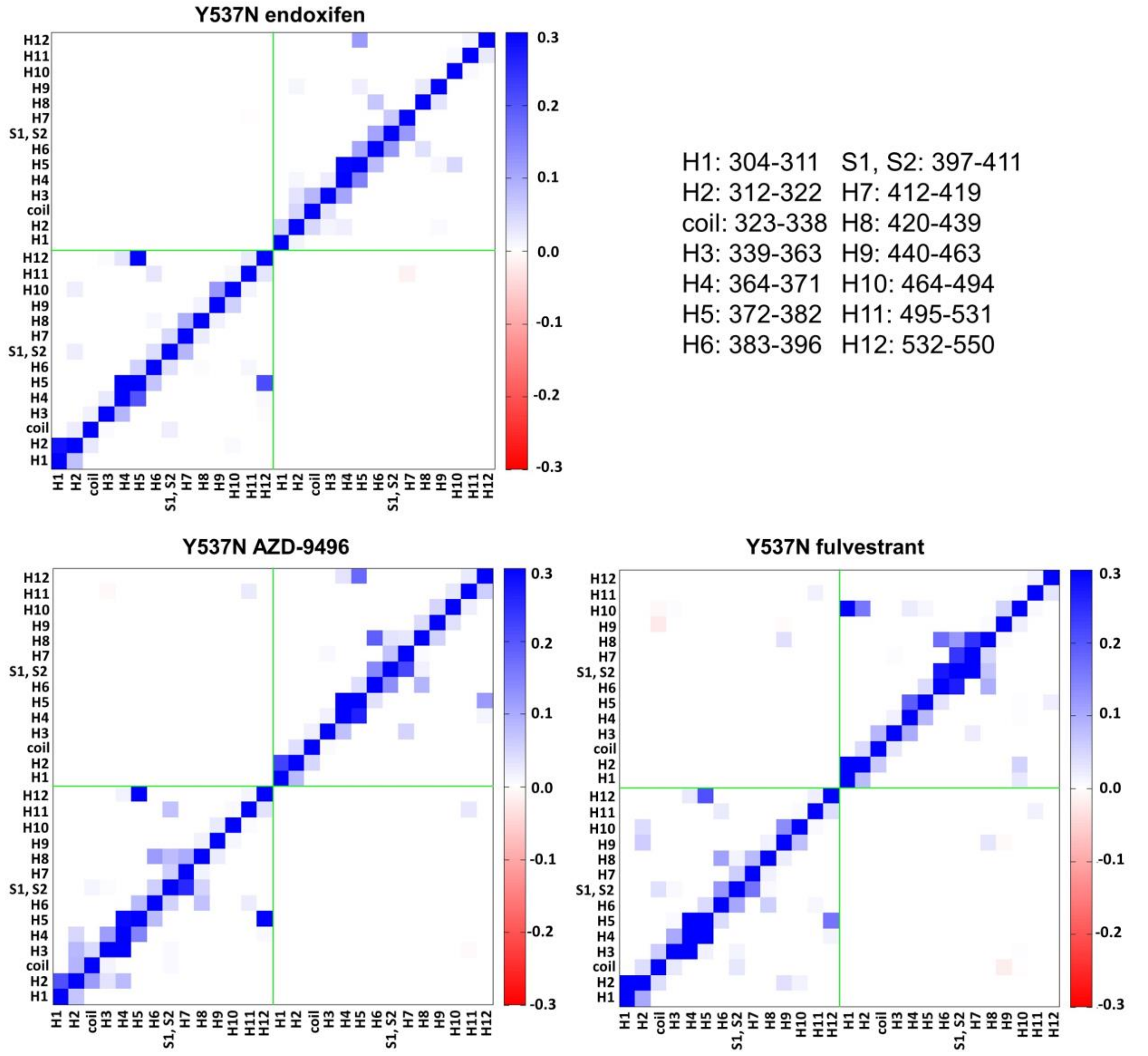

Figure S13. Per-column normalized cross-correlation maps of 14 regions (as defined in Fig. 1 of the main text) of both monomers of Y537N ERα in *antagonist*-bound form. The cross-correlation coefficients, normalized by columns, are reported in the range from -0.3 to 0.3 due to clarity reasons. Blue and red colours account for positive and negative correlation. Top left is END-bound ERα, bottom left AZD-9496-bound ERα, and bottom right FULV-bound ERα.

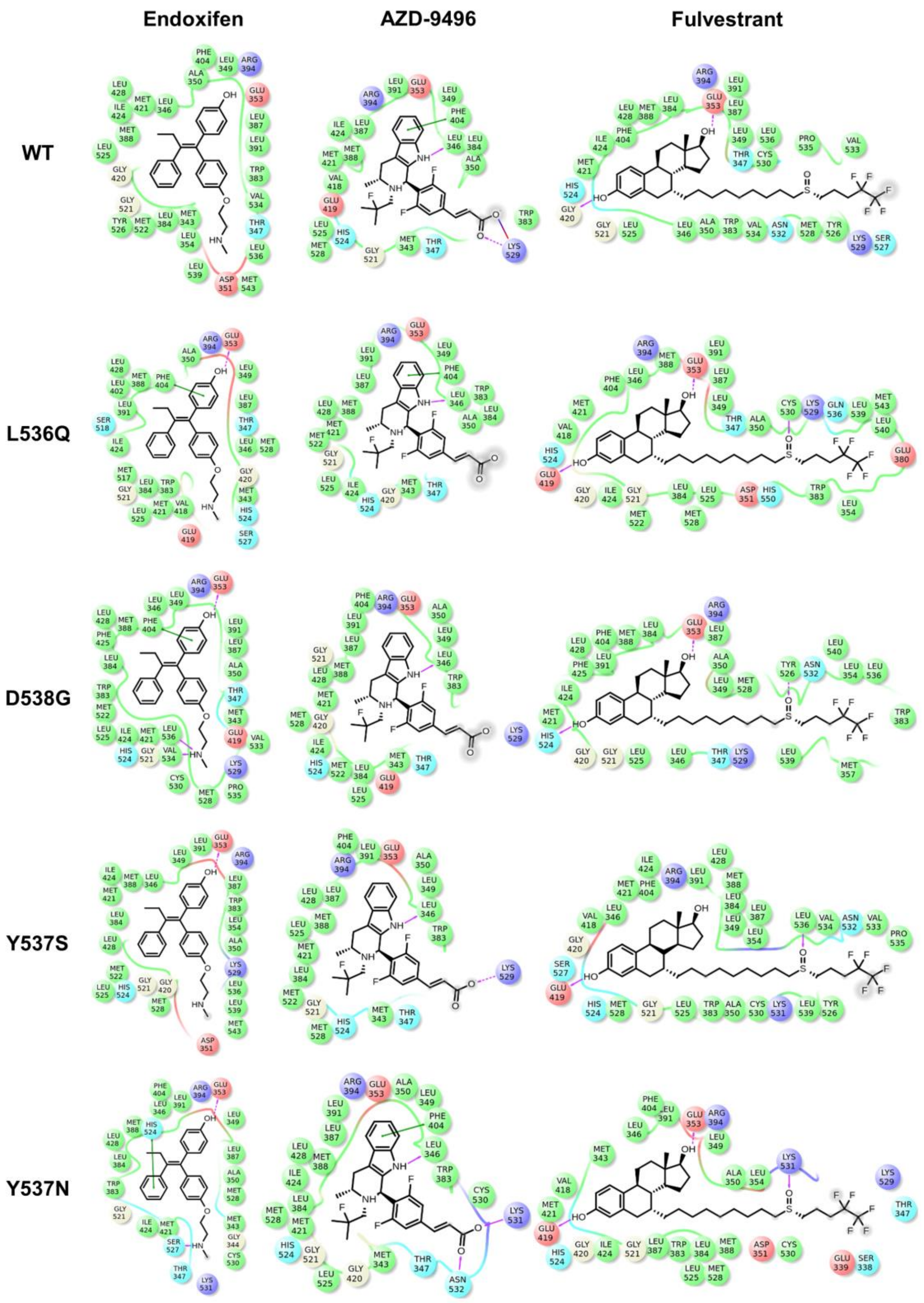

Figure S14a. 2D interaction maps of antagonists inside the binding pocket of ERα (monomer A). Green colour represents hydrophobic interactions, blue and red represent ionic interactions (negatively charged residues are coloured in red and positively charged in dark blue, respectively) and turquoise colour represents polar interactions. Glycines are coloured in bright yellow. Full magenta lines represent hydrogen bonds with the residues' backbone and the dashed magenta lines represent hydrogen bonds with the residues' side chains. Green lines show π-π interactions.

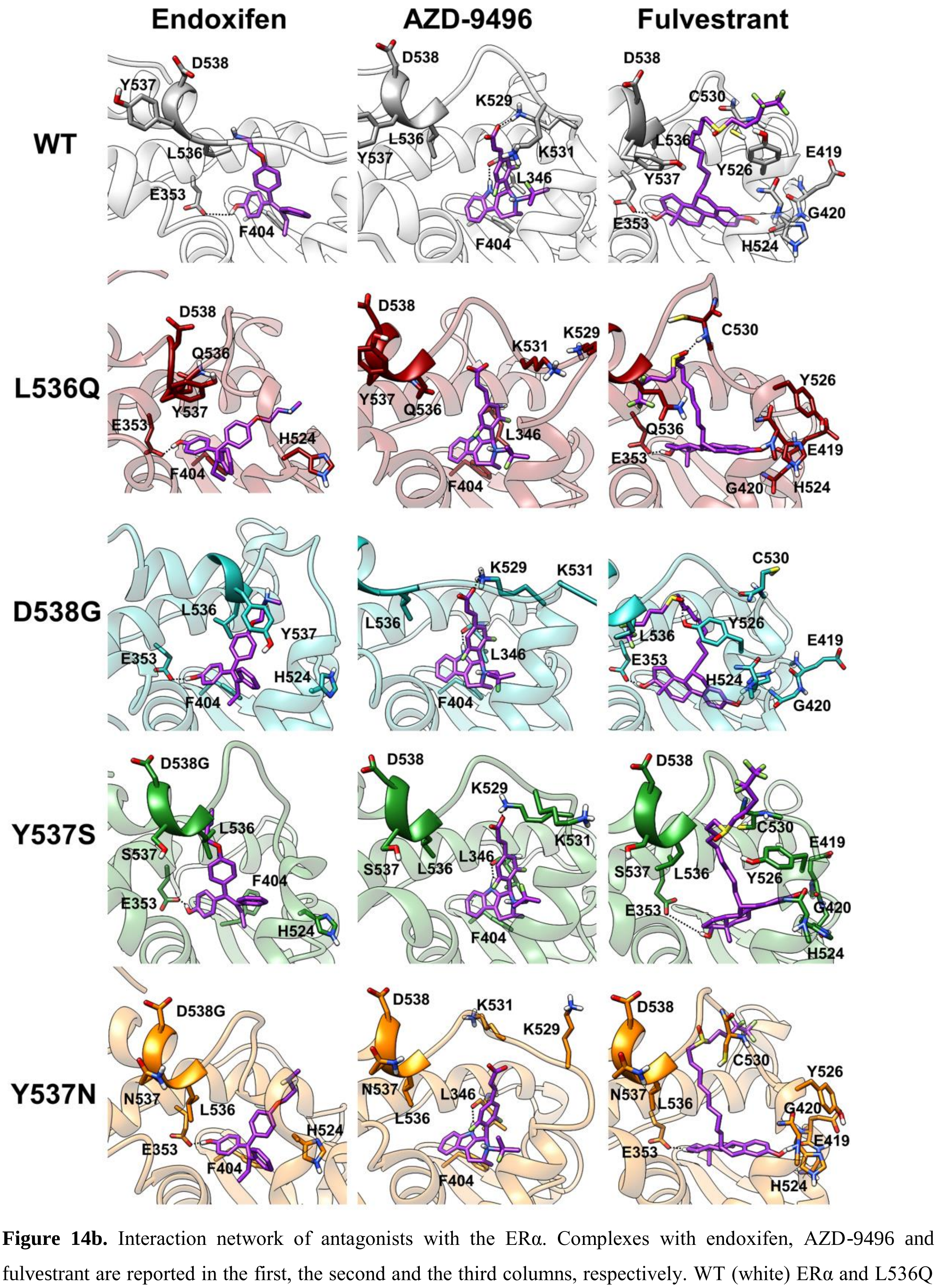

Figure 14b. Interaction network of antagonists with the ERα. Complexes with endoxifen, AZD-9496 and fulvestrant are reported in the first, the second and the third columns, respectively. WT (white) ERα and L536Q (dark red), D538G (turquoise), Y537S (green), and Y537N (orange) mERαs are reported from the top to the bottom rows. Endoxifen, AZD-9496, and fulvestrant are coloured in violet. H-bonds are shown as dotted black lines. Oxygen, nitrogen, and hydrogen atoms are depicted in red, blue, and white, respectively. The structures depict the highest populated clusters of monomer A as obtained from the clustering analysis.

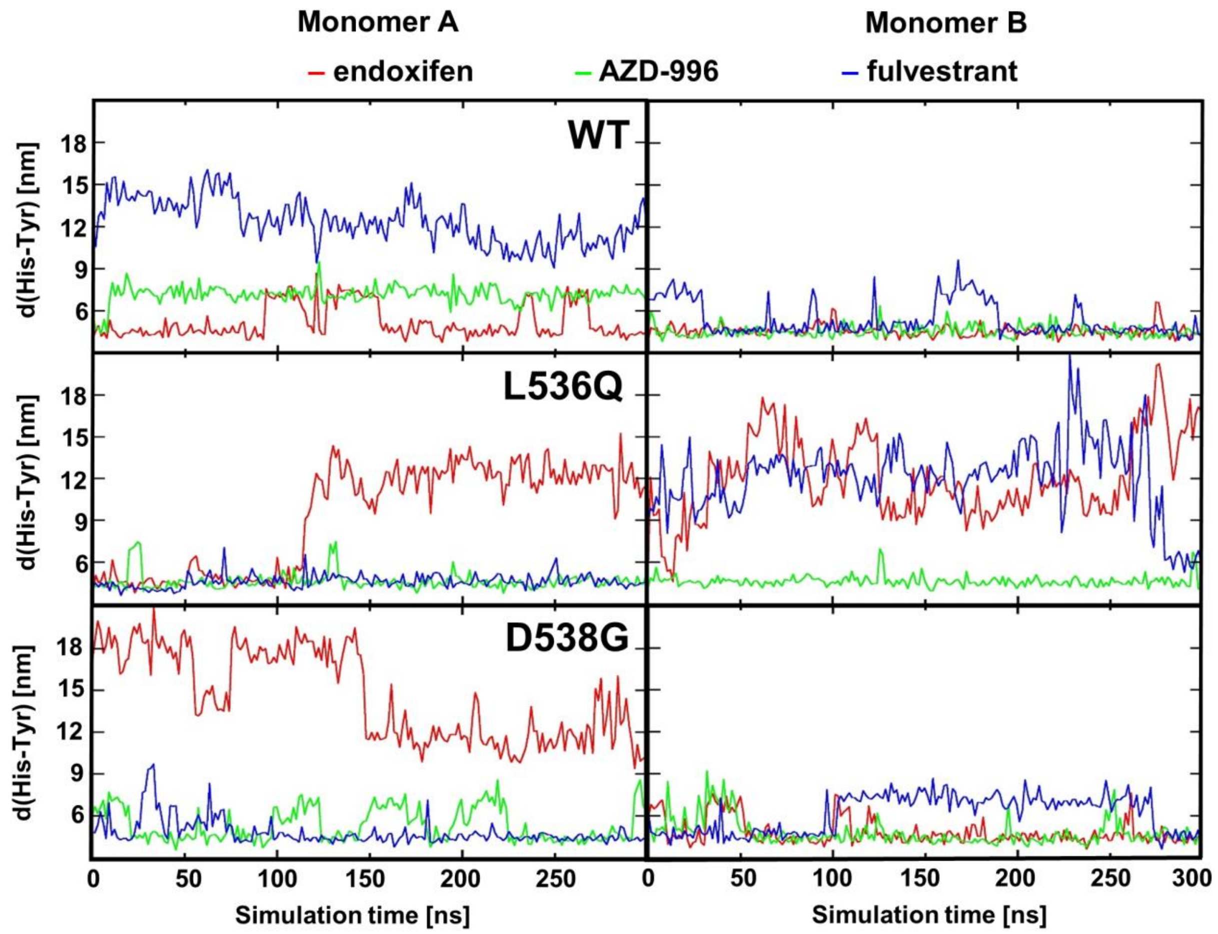

Figure S15. Distance between center of mass of Y537 and H377 rings for the analyzed 300 ns of classical MD simulations. Monomer A and B are shown in the left and right columns, respectively. END-, AZD-9496-, and FULV-bound complexes are shown in red, green, and blue, respectively.

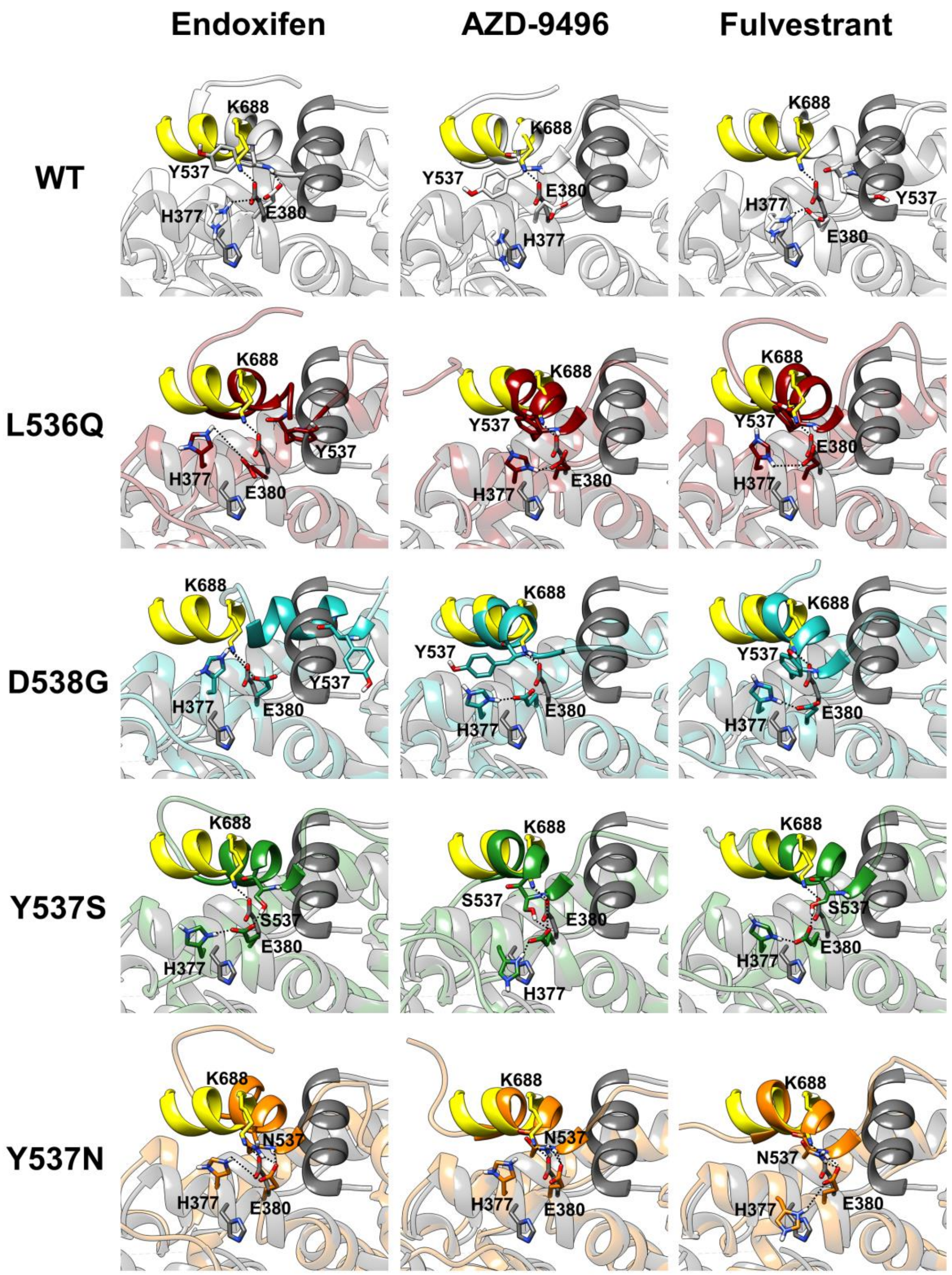

Figure S16. Most representative clusters obtained from the clustering analysis of separate antagonist-bound ERα monomers superimposed on the crystal structure of agonist-bound ERα (dark grey), with the coactivator protein located in the groove between H3 and H5 (yellow). In first column are END-bound complexes, in second AZD-9496-bound and in third FULV-bound ones. From the top to the bottom row WT (white) ERα, L536Q (dark red), D538G (turquoise), Y537S (green), and Y537N (orange) are shown. Hydrogen bonds are shown as dotted black lines. Oxygen, nitrogen, and hydrogen atoms are coloured in red, blue, and white, respectively. Only monomer A of each complex is shown.

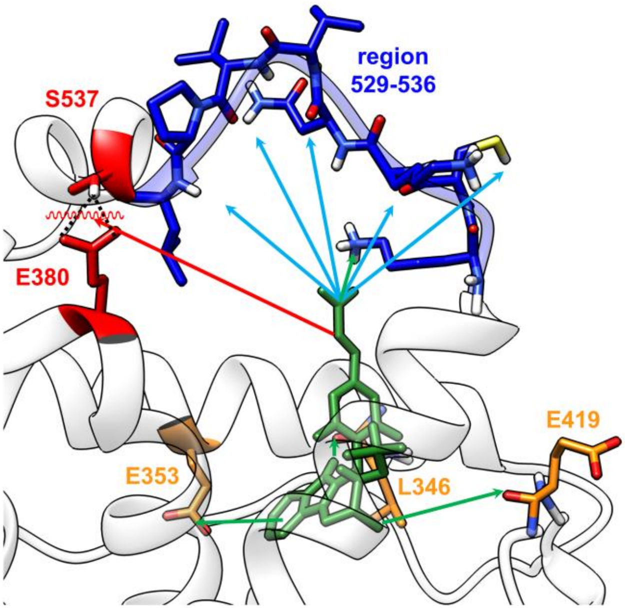

Figure S17. Regions of ERα that should be targeted by novel drugs effectively locking the antagonist mERαs. These should H-bond with the region 529-537 (blue), break H-bond between E380 and S/N537 (red) and form H-bonds with L346, E353 and E419 (orange) belonging to the ERα binding site. Figure is based on the highest populated cluster of Y537S ERα monomer A (white) with AZD-9496 (green) inside the binding site.

3. Supporting Tables

Table S1. Distribution of the highest populated cluster and first four highest populated clusters from the last 300 ns of classical MD simulations based the cut-off value of 0.275 nm. First and second columns of each section refer to monomer A and B, respectively.

	1st cluster		Total number of clusters	
WT				
Estrogen	100%	100%	1	1
Apo	100%	100%	1	1
Endoxifen	93.4%	95.1%	4	4
AZD-9496	98.9%	86.7%	3	3
fulvestrant	100%	78.7%	1	5
L536Q				
Estrogen	100%	100%	1	1
Apo	100%	100%	1	1
Endoxifen	57.3%	69.6%	5	4
AZD-9496	95.0%	100%	3	1
fulvestrant	80.1%	84.9%	5	3
D538G				
Estrogen	100%	100%	1	1
Apo	100%	98.6%	1	4
Endoxifen	100%	96.5%	1	4
AZD-9496	99.9%	99.6%	2	2
fulvestrant	98.7%	100%	3	1
Y537S				
Estrogen	99.9%	98.1%	2	3
Apo	98.9%	100%	3	1
Endoxifen	84.7%	97.1%	4	4
AZD-9496	100%	99.9%	1	2
fulvestrant	57.6%	100%	7	1
Y537N				
Estrogen	100%	98.0%	1	2
Apo	99.1%	97.4%	3	3
Endoxifen	99.8%	65.9%	2	4
AZD-9496	71.6%	73.0%	4	3
fulvestrant	53.8%	61.4%	9	7

Table S2. H-bond occurrence (%) between H3 residues (N348 and D351) and L11-12 residues (L536, Y/S/N537 – named Mut537, D538, L539, and L540) during the last 300 ns of MD simulation. First and second columns of each section refer to monomer A and B, respectively.

	N348 – L536 (Nδ2-H_2···O)		N351 – Mut537 (Oδ1/Oδ2···H-Oη (Tyr))[a] (Oδ1/Oδ2···H-Oγ (Ser))[b] (Oδ1/Oδ2···H_2-Nδ2 (Asn))[c] (Oδ1/Oδ2···H-N)[d]		D351 – N538 (Oδ1/Oδ2···H-N)		D351 – L539 (Oδ1/Oδ2···H-N)		D351 – L540 (Oδ1/Oδ2···H-N)	
WT										
apo		18.6%				96.0%		88.5%		92.4%
estrogen							3.3%	10.8%	6.2%	13.7%
L536Q										
apo					10.9%		10.7%		11.7%	
estrogen		3.0%*				46.0%	11.0%	73.2%	13.7%	80.9%
D538G										
apo			6.1%[a]	1.8%[a] 26.3%[d]	***	10.0%***		0.3%		2.4%
estrogen					25.3%***	***	36.8%	33.4%	46.6%	86.4%
Y537S										
apo	57.1%		63.6%[b]	85.6%[b]	99.4%		89.1%		91.3%	
estrogen	23.7%	16.8%	10.3%[b]	38.0%[b] 1.6%[d]	85.3%	73.7%	67.6%	60.0%	91.8%	73.7%
Y537N										
apo	81.9%			79.3%[c] 54.6%[d]**	105.6%		92.3%		88.3%	
estrogen		89.8%	80.5%[c]			105.8%		93.5%		81.8%

*Here L536 is mutated to Q536.

**Additional hydrogen bond is formed between R351 and R335 (Oδ1/Oδ2···H_2-Nη1/Nη2) with occurrence of 43.7%.

***Here D538 is mutated to G538.

Table S3. Occurrence of selected H-bonds (%) between H5 (H377 and E380) and H12 (L536, Y/S/N537 – named Mut537, and L540 along the last 300 ns of MD simulation.

	L536 – L540 (O···H-N)		E380 – Mut537 (Oε1/Oε2···H-N)		E380 – Mut537 (Oε1/Oε2···H-Nδ2/H-O)		E380 – H377 (Oε1/Oε2···H-Nδ1)	
WT								
Endoxifen	81.7%	73.2%	35.0%	7.9%			73.5%	81.9%
AZD-9496	84.8%	76.1%	8.4%	26.0%			4.4%	
Fulvestrant	30.0%	64.9%		3.0%			63.5%	82.4%
L536Q								
endoxifen	18.9%[*]	10.7%[*]					44.7%	62.1%
AZD-9496	57.4%[**]	58.7%[**]	17.4%	4.6%			57.6%	85.0%
fulvestrant	47.20%	36.6%[***]	8.4%	15.1%			62.7%	76.7%
D538G								
endoxifen	71.8%	85.7%		29.4%	18.8%		41.4%	82.4%
AZD-9496	79.6%	82.0%	10.0%	21.5%			35.3%	56.0%
fulvestrant	77.0%	82.4%	7.1%				69.6%	83.8%
Y537S								
endoxifen	78.5%	74.8%		35.3%	45.9%	71.9%	65.7%	60.8%
AZD-9496	82.0%	23.6%	61.5%		87.7%	[++]	17.1%	59.2%
fulvestrant	81.0%	42.2%[+++]	12.4%		37.4%	27.2%	34.2%	12.3%
Y537N								
endoxifen	85.5%	84.0%	50.4%	76.3%	90.3%	102.4%	22.3%	5.1%
AZD-9496	83.7%	81.3%	67.5%	69.7%	98.1%	102.8%	6.4%	16.1%
fulvestrant	71.4%	83.8%	26.7%	54.2%	70.7%	89.7%	22.2%	11.7%

[*]Additional hydrogen bond is formed between Y536 and W383 (Oε1···H-Nε1) with 12.2% and 31.4% occurrence in monomer A and B.

[**]Additional hydrogen bond is formed between Y536 and W383 (Oε1···H-Nε1) with 13.1% and 14.1% occurrence in monomer A and B.

[***]Additional hydrogen bond is formed between Y536 and L541 (O···H-N) with 53.9% in monomer B.

[++]Additional hydrogen bond is formed between S481 and W327 (Oγ···H-Nε1) with 31.4% in monomer A.

[+++]Additional hydrogen bond is formed between Y536 and L539 (O···H-N) with 36.8% in monomer B.

Table S4. Occurrence of H-bonds (%) during the last 300 ns of MD between antagonists and receptor (antagonist is always hydrogen bond acceptor).

	Ligand – Y526 (O···H-Oη)		Ligand – K529 (O···$H_3N\zeta$)		Ligand – C530 (O···H-N)		Ligand – K531 (O··· $H_3N\zeta$)'* (O··· H-N)"		Ligand – N532 (O···H-Nδ2)' (O··· H-N)"		Ligand – V534 (O···H-N)		Ligand – L536 (O···H-N)		Ligand – S537 (O···H-Oγ)' (O···H-N)"	
WT																
Endoxifen																
AZD-9496**			31.1%	29.7%												
Fulvestrant													46.3%			
L536Q																
Endoxifen																
AZD-9496**				33.4%												
fulvestrant				9.1%	66.9%							34.0%				
D538G																
endoxifen																
AZD-9496**			63.1%	44.6%												
fulvestrant	49.7%					48.5%										
Y537S																
endoxifen																
AZD-9496**			32.7%			22.1%	15.3%'							69.2%		38,0%'<br>36,1%"
fulvestrant									8.19%'				10.0%	71.6%		
Y537N																
endoxifen																
AZD-9496**		9.1%	19.0%	25.3%			35.7%"		6.7%"							
fulvestrant							12.7%"			10.9%"						

*Sum of hydrogen bond occurrences formed with the whole terminal amino group (Hζ1+Hζ2+Hζ3).

**Sum of hydrogen bond occurrences formed with both oxygen atoms of the ligand's carboxylic group.

Table S5. Occurrence of H-bonds (%) during the last 300 ns of MD between antagonists and receptor (antagonist is always hydrogen bond donor).

	L346 – ligand (O···H-N)		E353 – ligand (Oε1/Oε2···H-O)		E419 – ligand (O···H-O)		G420 – ligand (O···H-O)		S527 – ligand (O···H-N)	
WT										
endoxifen			82.7%	93.4%						57.6%
AZD-9496	83.9%	81.8%								
fulvestrant			82.3%	89.3%	52.9%	77.3%	21.4%	10.7%		
L536Q										
endoxifen			92.8%	80.4%					41.1%	
AZD-9496	69.1%	78.0%								
fulvestrant			69.9%	64.1%	73.1%	68.1%	6.5%	23.2%		
D538G										
endoxifen			67.2%	88.3%						
AZD-9496	71.2%	72.4%								
fulvestrant			84.5%	77.4%	*	79.2%		6.9%		
Y537S										
endoxifen			78.5%	86.7%						
AZD-9496	74.8%	79.6%								
fulvestrant			65.9%	95.0%	30.3%**	73.3%	23.1%	12.7%		
Y537N										
endoxifen			92.2%	37.2%					16.6%	
AZD-9496	80.6%	74.1%								
fulvestrant			95.4%	48.6%	91.7%	56.3%		27.0%		

*Here fulvestrant instead forms hydrogen bond with H524 (Nδ1···H-O) with occurence of 94.8%.

**Here fulvestrant forms additional hydrogen bond with H524 (Nδ1···H-O) with occurence of 23.2%.

Table S6. Interaction energies (kJ/mol) between relevant parts of the receptor (H5 and H6 against H12 and coil connecting H11 and H12) and the ligand during the analyzed 300 ns of molecular dynamics.

	(H5+H6)-(H12+loop)		(H5+H6)-ligand		(loop+H12)-ligand	
WT						
Endoxifen	-227.6±9.6	-201.4±6.7	-54.9±1.6	-36.5±1.3	-7.7±0.9	-45.4±2.1
AZD-9496	-208.0±3.6	-241.4±4.6	-49.3±1.0	-49.4±0.8	-91.7±9.4	-65.8±8.4
fulvestrant	-126.6±7.1	-200.9±7.5	-47.9±2.5	-46.6±2.8	-117.7±12.8	-69.9±13.0
L536Q						
endoxifen	-147.8±20.4	-157.5±10.4	-32.8±1.3	-47.6±1.1	-50.1±5.4	-43.6±7.4
AZD-9496	-227.8±6.7	-222.0±4.6	-47.6±1.2	-49.2±0.6	-16.9±12.1	-69.7±5.0
fulvestrant	-216.8±7.8	-219.4±11.0	-58.2±2.4	-84.5±3.9	-112.9±4.9	-93.9±13.6
D538G						
endoxifen	-112.6±9.1	-202.9±11.7	-41.2±1.4	-48.4±2.2	-66.2±3.3	-16.8±4.1
AZD-9496	-230.3±7.1	-203.8±11.7	-51.2±0.9	-50.7±1.2	-104.9±6.0	-75.0±10.8
fulvestrant	-220.2±4.5	-199.2±5.9	-66.3±1.9	-61.8±3.4	-42.7±2.6	-105.2±9.4
Y537S						
endoxifen	-196.0±8.1	-231.7±12.8	-52.8±1.3	-40.4±1.6	-35.0±5.8	-48.5±4.7
AZD-9496	-330.0±15.4	-145.4±7.8	-51.2±1.2	-57.6±3.0	-97.1±5.4	-196.3±28.2
fulvestrant	-193.3±31.2	-163.6±12.3	-70.6±10.4	-84.7±2.2	-85.1±11.6	-99.5±5.1
Y537N						
endoxifen	-277.1±13.7	-306.4±10.8	-39.0±1.3	-42.7±2.6	-34.6±1.7	-13.9±0.6
AZD-9496	-306.3±7.5	-289.6±7.5	-50.5±1.0	-48.6±1.5	-84.7±9.8	-54.4±13.1
fulvestrant	-228.4±17.4	-294.8±14.3	-42.1±1.7	-64.7±4.9	-88.6±16.1	-67.1±6.5

References

1 Fanning, S. W. *et al.* Estrogen receptor alpha somatic mutations Y537S and D538G confer breast cancer endocrine resistance by stabilizing the activating function-2 binding conformation. *eLife* **5**, (2016).

2 Merenbakh-Lamin, K. *et al.* D538G mutation in estrogen receptor-alpha: A novel mechanism for acquired endocrine resistance in breast cancer. *Cancer Res.* **73**, 6856-6864, (2013).

3 Palermo, G., Miao, Y., Walker, R. C., Jinek, M. & McCammon, J. A. Striking plasticity of CRISPR-Cas9 and key role of non-target DNA, as revealed by molecular simulations. *ACS Cent. Sci.* **2**, 756-763, (2016).

4 Pettersen, E. F. *et al.* UCSF Chimera--a visualization system for exploratory research and analysis. *J. Comput. Chem.* **25**, 1605-1612, (2004).
